# COVID-19-related medical research: a meta-research and critical appraisal

**DOI:** 10.1186/s12874-020-01190-w

**Published:** 2021-01-04

**Authors:** Marc Raynaud, Huanxi Zhang, Kevin Louis, Valentin Goutaudier, Jiali Wang, Quentin Dubourg, Yongcheng Wei, Zeynep Demir, Charlotte Debiais, Olivier Aubert, Yassine Bouatou, Carmen Lefaucheur, Patricia Jabre, Longshan Liu, Changxi Wang, Xavier Jouven, Peter Reese, Jean-Philippe Empana, Alexandre Loupy

**Affiliations:** 1 Paris Translational Research Epidemiology and Biostatistics Department, Hôpital Necker, 149 rue de Sèvres, 75015 Paris, France; 2 The First Affiliated Hospital of Sun Yat-Sen University, Guangzhou, China; 3 Montpellier University Hospital, Montpellier, France; 4 Pitié-Salpêtrière University Hospital, Assistance Publique-Hôpitaux de Paris, Sorbonne University, Paris, France; 5 Paediatrics Unit, Necker University Hospital, Paris, France; 6 Immunology and Nephrology Department, Saint Louis Hospital, Paris, France; 7 Cochrane Pre-hospital and Emergency Care, Necker University Hospital, Paris, France; 8 University of Pennsylvania School of Medicine, Philadelphia, PA USA

**Keywords:** COVID-19, Systematic review, Critical appraisal, Quality of research

## Abstract

**Background:**

Since the start of the COVID-19 outbreak, a large number of COVID-19-related papers have been published. However, concerns about the risk of expedited science have been raised. We aimed at reviewing and categorizing COVID-19-related medical research and to critically appraise peer-reviewed original articles.

**Methods:**

The data sources were Pubmed, Cochrane COVID-19 register study, arXiv, medRxiv and bioRxiv, from 01/11/2019 to 01/05/2020. Peer-reviewed and preprints publications related to COVID-19 were included, written in English or Chinese. No limitations were placed on study design. Reviewers screened and categorized studies according to *i)* publication type, *ii)* country of publication, and *iii*) topics covered. Original articles were critically appraised using validated quality assessment tools.

**Results:**

Among the 11,452 publications identified, 10,516 met the inclusion criteria, among which 7468 (71.0%) were peer-reviewed articles. Among these, 4190 publications (56.1%) did not include any data or analytics (comprising expert opinion pieces). Overall, the most represented topics were infectious disease (*n* = 2326, 22.1%), epidemiology (*n* = 1802, 17.1%), and global health (*n* = 1602, 15.2%). The top five publishing countries were China (25.8%), United States (22.3%), United Kingdom (8.8%), Italy (8.1%) and India (3.4%). The dynamic of publication showed that the exponential growth of COVID-19 peer-reviewed articles was mainly driven by publications without original data (mean 261.5 articles ± 51.1 per week) as compared with original articles (mean of 69.3 ± 22.3 articles per week). Original articles including patient data accounted for 713 (9.5%) of peer-reviewed studies. A total of 576 original articles (80.8%) showed intermediate to high risk of bias. Last, except for simulation studies that mainly used large-scale open data, the median number of patients enrolled was of 102 (IQR = 37–337).

**Conclusions:**

Since the beginning of the COVID-19 pandemic, the majority of research is composed by publications without original data. Peer-reviewed original articles with data showed a high risk of bias and included a limited number of patients. Together, these findings underscore the urgent need to strike a balance between the velocity and quality of research, and to cautiously consider medical information and clinical applicability in a pressing, pandemic context.

**Systematic review registration:**

https://osf.io/5zjyx/

**Supplementary Information:**

The online version contains supplementary material available at 10.1186/s12874-020-01190-w.

## Background

Originally reported in the Hubei province of China, the coronavirus disease 2019 (COVID-19) represents a serious and pressing threat to health all around the world [1]. As of November 30th, 2020, a total of 1,461,049 deaths among 62,829,641 cases were confirmed [2]. Since the outbreak started, a huge worldwide effort has been launched to address the unmet need for improving diagnosis, understanding the determinants, prognosis, pathogenicity of COVID-19 infection, and thereby optimizing decision-making and patient management, therapeutics and prevention of the disease [3].

In this context, while health systems are still adjusting to the pandemic situation, medical research and peer-review process have shown an unprecedented acceleration to ease scientific communication [4] with many topics around COVID-19 covered [5–7].

Despite the vast investment by government agencies and private consortiums to trace the number of confirmed COVID-19 cases and related deaths in real-time, and efforts to share data worldwide, concerns about the risk of expedited science have been raised [8–10]. So far, although numerous investigations have been conducted, many have shown suboptimal design, methods, analytics and interpretation. Some articles were not submitted for peer-review and have been strongly criticized [11, 12], and some were withdrawn after direct consequences on public health [13]. Furthermore, it has been pointed out that a substantial number of published articles was composed of expert opinion without original data and analytics [14–16]. Last, among articles with data, concerns have been expressed about their methodology and asymmetry between scientific content and claims for utility [11, 17].

Hence, in this context encouraging open-access research [18], preprints [19], and expedited review by medical journals [20], the sharp increase of COVID-19-related publications may sometimes result in flawed, biased, or misleading research. Together these phenomena run the risk of promoting incorrect information and biased clinical practice, thereby hampering appropriate decision-making and potentially harming patients [10, 21]. In addition, this trend may have unfortunate consequences on public health policies and future research, thus delaying the generation of valid scientific insights that can enhance patient management and treatment discovery.

Providing a holistic and systematic appraisal of COVID-19 research in the current pandemic context is an unmet need. To achieve this goal, we designed a meta-research including all available COVID-19 literature using a large task force dedicated to high volume articles analytics. We aimed at investigating the dynamics of COVID-19 publications, assessing the type of medical articles published and the related health topic, and critically appraising the peer-reviewed, original articles.

## Methods

### Search strategy

We followed the Preferred Reporting Items for Systematic Reviews and Meta-Analyses (PRISMA) [22] statement to design and report our meta-research, where applicable (supplementary methods 1). A systematic literature search was performed in Pubmed and Cochrane COVID-19 study registry for peer-reviewed medical articles. Additional search using bioRxiv, medRxiv, and arXiv platforms was performed to include preprints for additional analysis. The literature search was performed between 1 November 2019 and 1 May 2020. “COVID-19”, “SARS-CoV-2” and their synonyms were used for the searches. The detailed search strategy for each database is provided in supplementary methods 2. The protocol of the study is available at https://osf.io/5zjyx/.

### Inclusion and exclusion criteria

Any medical publication related to COVID-19 was included. No limitations were placed on publication type and study design. Both peer-reviewed and preprints articles were included. Publication related to protocol reporting and full-text unavailable were excluded. The language was limited to English and Chinese because China is the first country to report COVID-19 cases and because the majority of high impact scientific publications are published in the English language.

### Screening and data extraction

First, after duplicate elimination, the references were screened based on the titles and abstracts by two reviewers (HZ, JW). To ensure accuracy, a pilot exercise was conducted using the same 100 articles to calibrate the process of reviewer assessment of studies before the format screening process commenced. Any discrepant result was discussed by the two reviewers and resolved by consensus, or where necessary, through adjudication by a third reviewer. Subsequently, the references that might meet the inclusion criteria were selected for full-text reading. Before full-text reading, all reviewers were trained using the same 100 full-texts. The final set of publications were then randomly divided and assigned to eight reviewers trained in systematic review and meta analyses (ZD, QD, VG, KL, MR, JW, YW and HZ). Finally, all excluded references were re-checked by two reviewers (MR, AL).

The following data from each included article were extracted: (1) study basic information: journal, title, publication date, family name and country of first author, (2) categorization index: type of publication and topics. For the original research articles that underwent quality assessment, the following information were recorded: number of patients, primary and secondary outcome, patient consent and 151 total items related to the quality assessment tools (see dedicated chapter in the methods section).

Endnote (Endnote X9, Thomson Reuters, Philadelphia, PA, USA), Excel (Excel 2019, Microsoft, Redmond, WA, USA) and NoteExpress (Version 3.2, Beijing Aegean Software Co., Ltd., Beijing, China) software were used for the screening, categorization, and appraisal of medical articles.

### Data analysis

#### Dynamics of COVID-19 publications

We aimed at investigating the dynamics of COVID-19 publications. To do so, we recorded the day of publication for each publication, and used the *smooth* function from *stats* R package, to represent the cumulative numbers of medical articles according to the time of publication. The number of COVID-19 confirmed cases worldwide was extracted from the publicly available database of the University of John Hopkins: COVID-19 Map - Johns Hopkins Coronavirus Resource Center [23].

#### Categorization of publications

We aimed at categorizing the included medical articles by type of publications and related topics. Six types of publication were pre-specified following definitions of BMC Medical Research Methodology: original article, research letter, review, systematic review, case reports or case series, and publication without original data (gathering viewpoint, editorial, perspective, expert opinions). To categorize the topics of all included articles, an original list of topics was developed before the review started (supplementary Table 1). Each study was categorized in up to three topics.

#### Critical appraisal of original articles

We aimed at critically appraising the peer-reviewed original articles, if they met the following criteria: (1) clinical studies involving human subjects, (2) modelling and simulation studies based on public health open access data, e.g. epidemiological models aimed at understanding the spread of the disease and the impact of different interventions. Systematic reviews and basic science studies including animal, in-vitro and bioinformatic studies were excluded. Different quality assessment tools were used based on the study design (supplementary methods 3). To minimize errors, all the reviewers were trained on several articles to use each assessment tool in a standardized manner. Uncertainty was resolved through daily discussion among reviewers.

Validated assessment tools were used to assess the quality of COVID-19 original articles according to their respective design: *i)* The New Ottawa Scale (NOS) tool for case-control studies and cohort studies [24]. *ii)* The Cochrane risk-of-bias (RoB 2) tool used for evaluating randomized controlled trials [25]. *(iii)* The Risk Of Bias In Non-randomised Studies - of Interventions (ROBINS-I) tool was used for assessing non-randomized interventional studies [26]. *(iv)* The Meta Quality Appraisal Tool (MetaQAT) was used for assessing simulation-based studies [27]. *(v)* (AXIS) tool was used for cross-sectional studies [28]. *(vi)* The Quality Assessment of Diagnostic Accuracy Studies (QUADAS-2) tool was used for evaluating diagnostic study [29]. *(vii)* The Quality in Prognostic Studies tool (QUIPS) [30] was used for assessing prognostic studies, *(viii)* The checklist from Cochrane Murad et al. [31] was used for assessing case series. Details about the assessment tools used for each study type is presented in supplementary method 3 and supplementary Tables 2, 3, 4, 5, 6, 7, 8, 9, 10.

R (version 3.2.1, R Foundation for Statistical Computing) and STATA (version 14, Data Analysis and Statistical Software) software were used for the data analyses.

## Results

### Identification and categorization of COVID-19 related publications

**Fig. 1:**
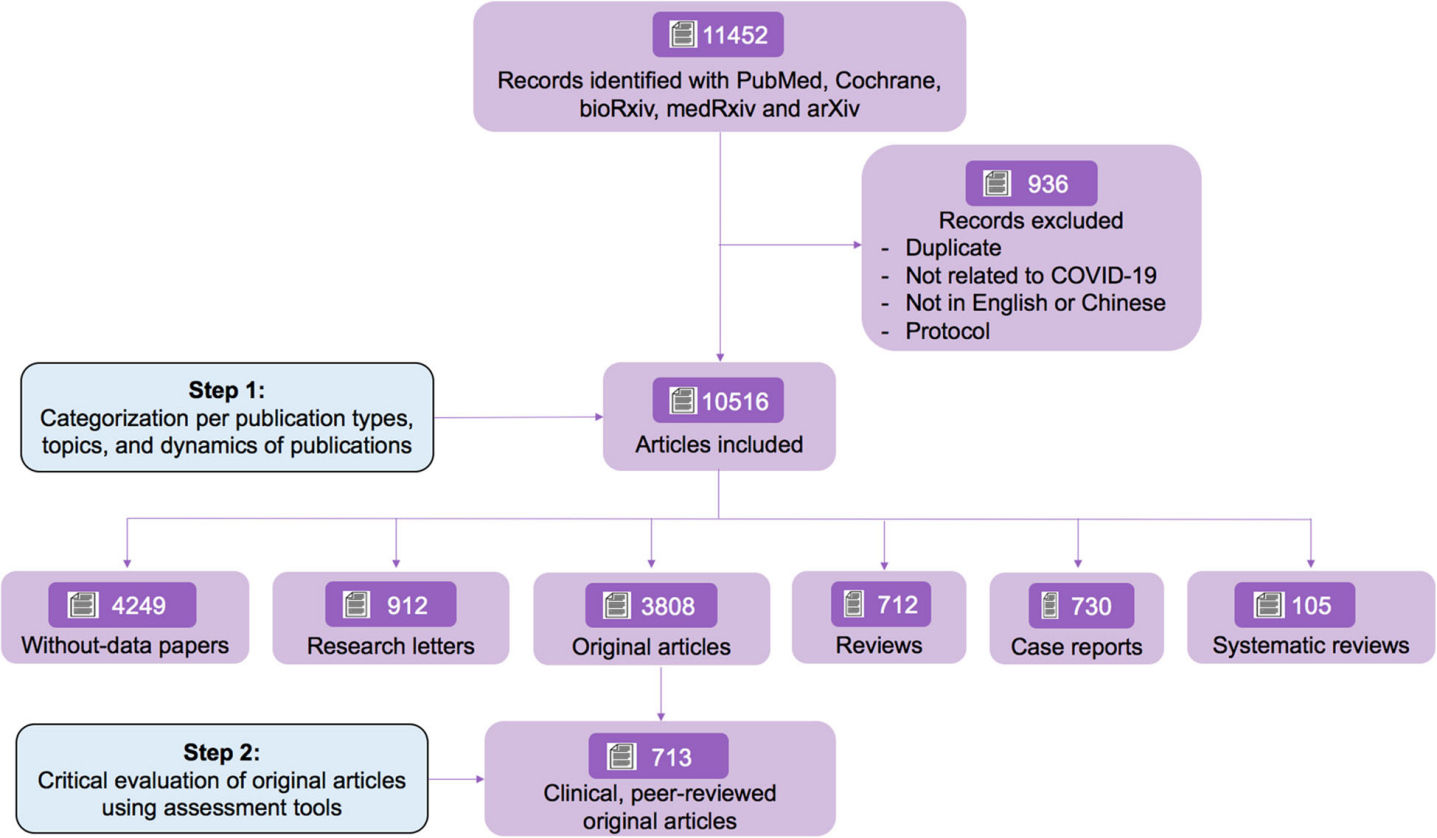
Study flowchart. Flowchart based on preferred reporting for systematic reviews and meta-analyses (PRISMA) guidelines (supplementary method 1), depicting the review process and the inclusion/exclusion criteria. Pubmed and Cochrane COVID-19 register study were used for identifying peer-reviewed articles, and bioRxiv, medRxiv and arXiv were used for identifying preprints

A total of 11,452 peer-reviewed or preprints references made available from 1 November 2019 to 1 May 2020 have been identified with our search strategy. After removing duplicates, studies not related to COVID-19, studies written in a language different than English or Chinese, and protocols, 10,516 references remained of which 7468 (71.0%) were peer-reviewed articles. Among these, 4190 (56.1%) articles were opinions that did not include any data or analytics (comprising viewpoints, editorials, perspectives, expert opinions). Instead, original studies accounted for 1109 articles (14.9%), case reports 697 (9.3%), research letters 786 (10.5%), reviews 638 (8.5%) and systematic reviews 48 (0.6%). The flowchart of the study is presented in Fig. 1. The distribution of the type of publication is depicted in supplementary Figure 1.

### Dynamics of COVID-19 publication and worldwide distribution

**Fig. 2:**
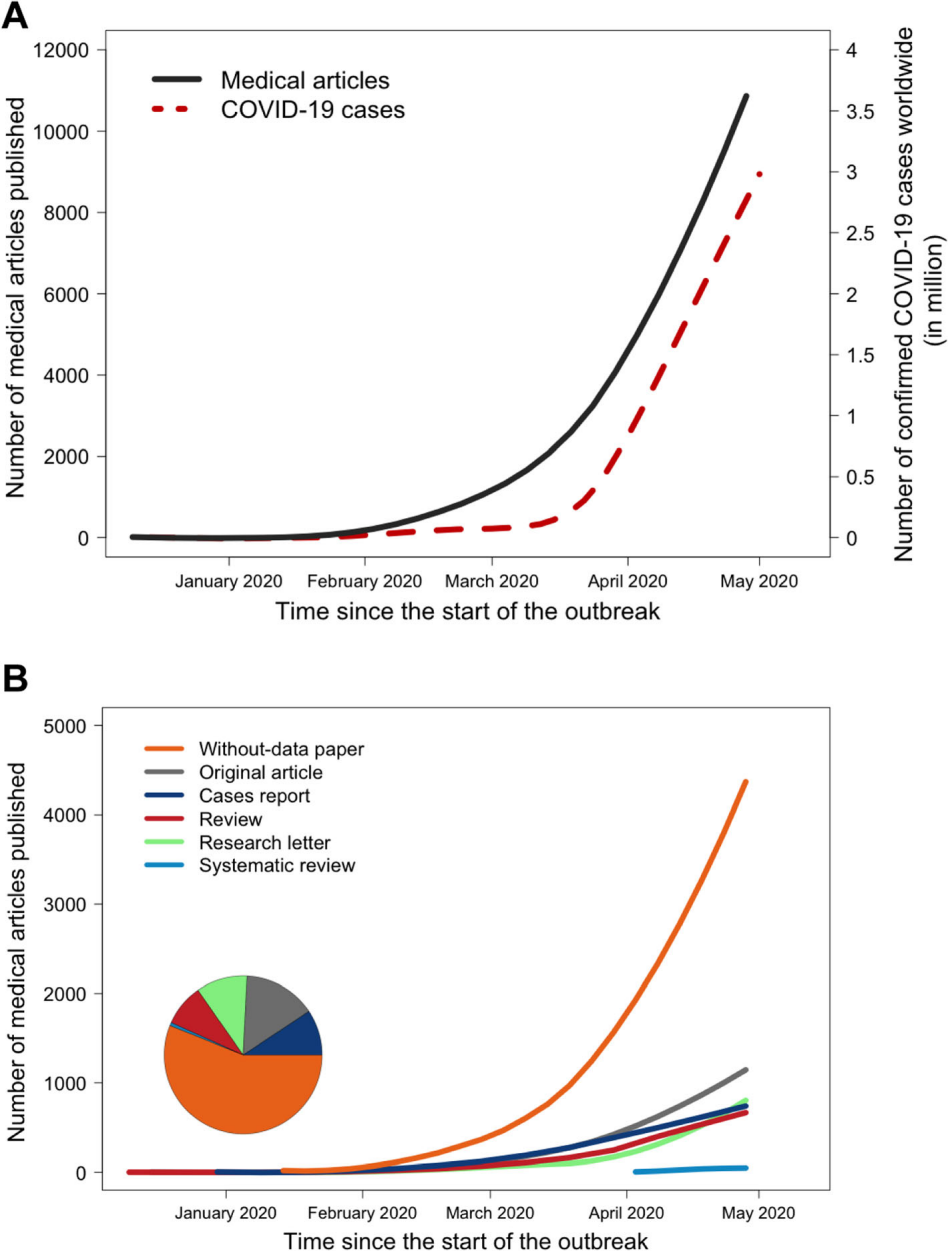
Dynamics of publication and trends in COVID-19 spread over time. This figure depicts the number of medical articles published and number of COVID-19 cases diagnosed worldwide over time (panel **a**) and the trends over time of peer-reviewed articles (*N* = 7468, panel **b**), categorized into publications without original data (*N* = 4190, 56.1%), original articles (*N* = 1109, 14.9%), cases reports (*N* = 697, 9.3%), reviews (*N* = 638, 8.6%), research letters (*N* = 786, 10.5%), and systematic reviews (*N* = 48, 0.6%)

COVID-19 related medical publication showed exponential growth since February 2020 with 203.3 ± 48.2 articles published every week in February 2020 up to 1645.0 ± 542.1 in April 2020 (Fig. 2a) with peer-reviewed articles displaying a more dramatic increase than preprint articles (supplementary Figure 2). In peer-reviewed medical studies, publications without original data dominated the exponential growth of COVID-19 literature with 261.9 ± 61.1 articles published every week (Fig. 2b), followed by original articles, case reports, reviews, research letters and systematic reviews with 69.3 ± 22.3, 43.2 ± 9.0, 39.9 ± 11.9, 49.3 ± 9.9, and 3.0 ± 2.5 articles respectively published every week (Fig. 2b).

In preprints, original articles represented 2699 (88.5%) of articles, reviews 74 (2.4%), publications without original data 59 (1.9%), case reports 33 (1.1%), research letters 126 (4.1%) and systematic reviews 57 (1.9%) (supplementary Figures 1C and 2).

**Fig. 3:**
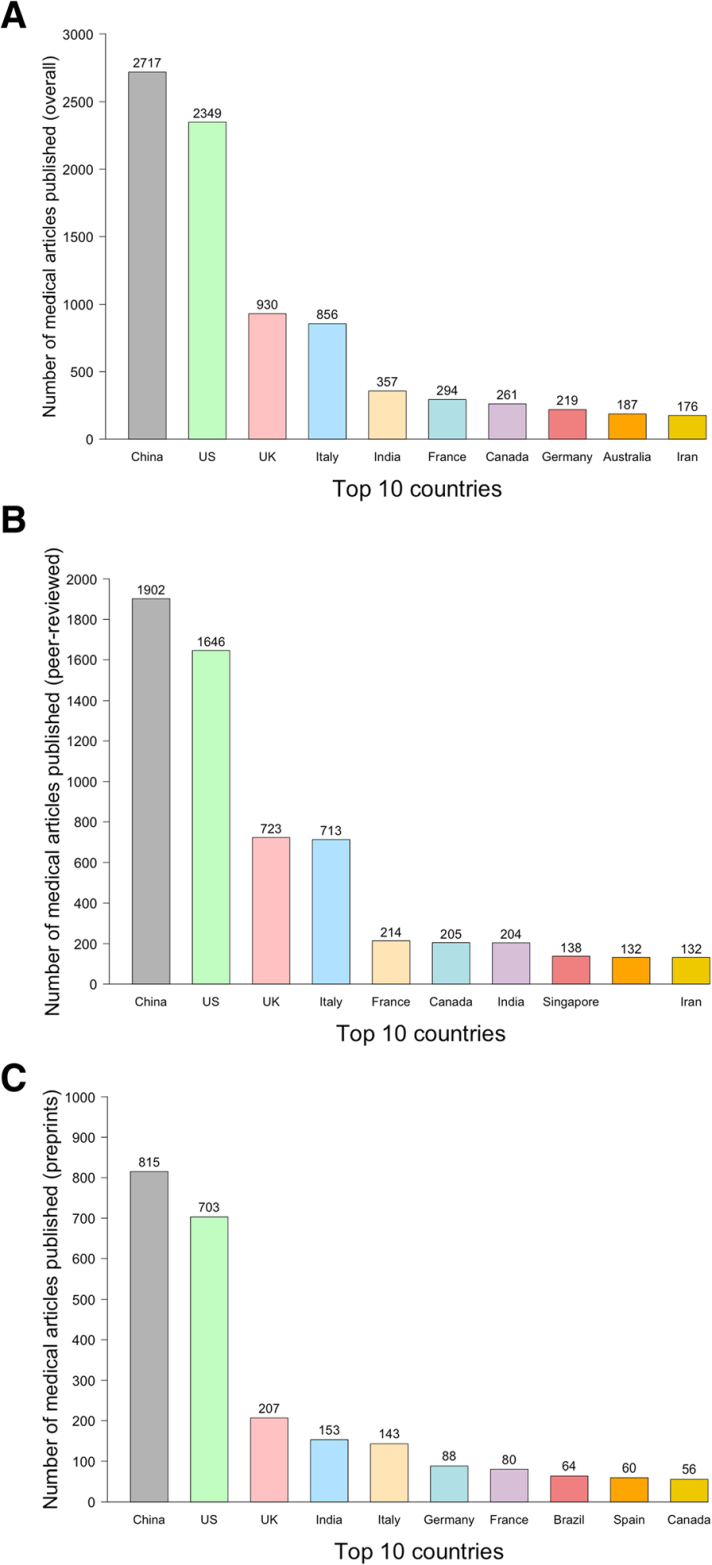
Number of COVID-19-related medical articles published by authors from 10 most productive countries. **a** All; **b** Peer-reviewed articles; **c** Preprints

We then assessed the distribution of countries among all COVID-19 publications. The top five COVID-19 publishing countries were China with 2717 (25.8%) studies, the United States 2349 (22.3%), United Kingdom 930 (8.8%), Italy 856 (8.1%) and India 357 (3.4%), followed by France 294 (2.8%), Canada 261 (2.5%), Germany 219 (2.1%), Australia 187 (1.8%) and Iran 176 (1.7%) (Fig. 3). We present the dynamics of COVID-19 publications according to the number of confirmed COVID-19 cases for each country in the supplementary Figure 3.

### COVID-19 publications and related topics

**Fig. 4:**
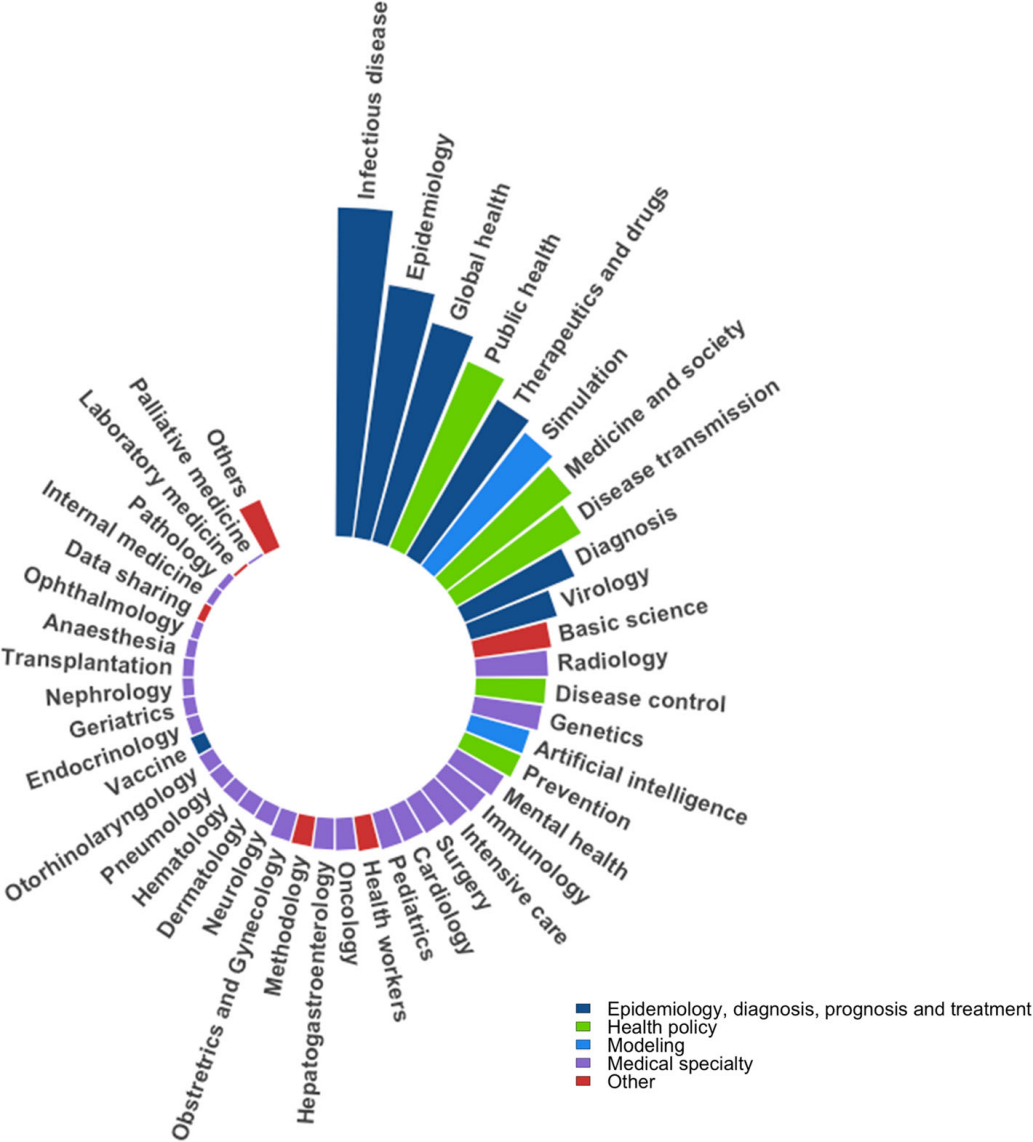
Topics addressed in COVID-19-related medical articles. Each barplot represents the number of articles dealing with the corresponding topic. 200 topics were listed after the first screening. After the discussion and consensus with our teams, they were categorized into 45 topics presented in the figure. All topics meaning is detailed in the supplementary Table 1. For a better insight, we have defined an ultimate categorization, defined in the legend: 1) Epidemiology, diagnosis, prognosis and treatment, 2) Health policy, 3) Modeling, 4) Medical specialty, and 5) Other

Topics related to COVID-19 publications are depicted in Fig. 4. Overall, among the 10,516 articles included in this study, 45 topics have been identified. The classification scheme is presented in detail in the supplementary Table 1.

The top five most represented topics were 1) infectious disease (*n* = 2326, 22.1%), 2) epidemiology (*n* = 1802, 17.1%), 3) global health (*n* = 1602, 15.2%), 4) public health (*n* = 1426, 13.6%), and 5) therapeutics, drugs and medicines (*n* = 1277, 12.1%).

A total of 1193 articles (11.3%) related to simulation-based studies, and 1124 (10.7%) and 1020 (9.7%) articles respectively related to medicine and society and disease transmission and have also been identified. Remaining categories were mainly related to medical specialties studying the COVID-19 specific impact on different organs (lung, heart, cardiovascular system, cerebral, kidney, etc.). The distribution of topics and medical specialties in peer-reviewed and preprint articles separately is depicted in supplementary Figures 4 and 5.

### Critical appraisal of original articles

We assessed the quality of research of the 713 clinical, peer-reviewed original articles (i.e. excluding preprints), comprising observational and interventional studies. We used a total of 9 validated tools totaling 151 items to address all types of study. The datasets corresponding to these analyses can be downloaded at https://www.paristransplantgroup.org/covid-19-related-medical-research.html. The detail of the assessment tools used for each study type is presented in supplementary method 3. Basic characteristics of these studies are presented in Table 1. The detailed assessment of study quality according to study type is shown in supplementary Figures 6, 7, 8 and 9.

**Table 1:** Basic characteristics of COVID-19-related, peer-reviewed original articles

Type of original article studies involving patients	Number of studies (%)	Number of patients Median (IQR)	Number of studies at risk of bias (%)			Patient consent			
			Low	Intermediate	High	Written informed consent N (%)	Oral consent N (%)	Open data N (%)	No consent N (%)
**Case-control**	68 (9.5)	108 (62–211)	11 (16.2)	25 (36.7)	32 (47.1)	22 (32.4)	2 (2.9)	2 (2.9)	42 (61.8)
**Cohort**	50 (7.0)	110 (54–327)	7 (14.0)	20 (40.0)	23 (46.0)	15 (30.0)	1 (2.0)	4 (8.0)	30 (60.0)
**Cross-sectional**	306 (42.9)	217 (80–730)	10 (3.3)	43 (14.0)	253 (82.7)	89 (29.1)	18 (5.9)	75 (24.5)	112 (40.5)
**Case series**	129 (18.1)	18 (9–53)	9 (6.9)	26 (20.2)	94 (72.9)	24 (18.6)	15 (11.6)	3 (2.3)	87 (67.4)
**Diagnostic**	37 (5.2)	84 (49–215)	0 (0)	0 (0)	37 (100.0)	3 (8.1)	0 (0)	0 (0)	34 (91.9)
**Prognostic**	8 (1.1)	143 (66–217)	3 (37.5)	1 (12.5)	4 (50.0)	0 (0)	0 (0)	0 (0)	8 (100.0)
**Simulation**	185 (25.9)	1428 (14–40,696)	16 (8.6)	47 (25.4)	122 (66.0)	3 (1.6)	1 (0.5)	131 (70.8)	50 (27.0)
**Non-randomized trial**	8 (1.1)	35 (29–58)	1 (12.5)	1 (12.5)	6 (75.0)	4 (50.0)	0 (0)	0 (0)	4 (50.0)
**Randomized controlled trial**	4 (0.6)	56 (29–111)	0 (0)	2 (50.0)	2 (50.0)	2 (50.0)	0 (0)	0 (0)	2 (50.0)

This table displays the basic characteristics of the 713 clinical, peer-reviewed, COVID-19-related, original articles we critically appraised based on several risk of bias tools, according to the type of studies. Eighty-two studies were assessed using two tools, to better reflect their design. Shown are the number of studies, the median number of patients, the overall risk of bias after quality assessment, and how patient consent was addressed by authors

Among original articles, cross-sectional studies (*N* = 306, 42.9%) and simulation-based studies (*N* = 185, 25.9%) were the most represented, followed by case series (*N* = 129, 18.1%), case-control studies (*N* = 68, 9.5%), cohort studies (*N* = 50, 7.0%), and diagnostic studies (*N* = 37, 5.2%) (supplementary Figure 6). Interventional non-randomized trials, prognostic studies and randomized controlled trials accounted for 8 (1.1%), 8 (1.1%) and 4 (0.6%) respectively and are presented in supplementary Figures 7, 8 and 9.

Among the 306 cross-sectional studies, the median number of patients was 217 (IQR = 80–730). A total of 253 studies (82.7%) were at high risk of bias according to the AXIS tool checklist, mostly driven by lack of justification in the sample size (55.9%), and the selection bias due to the low completion rate (59.3%).

Among the 185 COVID-19 simulation-based studies, the median number of patients was of 1428 (IQR = 14–40,696). A total of 122 (65.9%) studies showed high risk of bias according to the MetaQAT tool, with 74.1 and 36.8% for the methods and findings respectively.

Among the 129 original articles with case series data, the median number of patients was 18 (IQR = 9–53), and 94 (72.9%) were at high risk of bias according to the checklist from Murad et al. [31]. The follow-up duration was inadequate for 51 studies (39.5%). Twenty-seven studies (20.9%) did not provide sufficient data description while 38 (29.5%) lacked patients representativeness.

Among the 68 case-control studies, the median number of patients was 108 (IQR = 62–212), and 32 (47.1%) were considered at high risk of bias according to the New Ottawa Scale (NOS). Case-control studies displayed a median NOS score of 7.0 (IQR = 5.0–8.0). The selection items displayed a more biased score, as compared with comparability and exposure items.

Among the 50 cohort studies, a total of 23 (46.0%) studies were considered at high risk of bias according to the NOS scale. The median number of patients was of 110 (IQR = 54–327). Cohort studies displayed a median NOS score of 7.0 (IQR = 5.5–8.0). The comparability items displayed a more biased score, as compared with selection and exposure items.

Among the 37 COVID-19 diagnostic studies, the median number of patients was of 84 (IQR = 49–215), and all showed a high risk of bias according to the QUADAS-2 tool. These included patient selection (*N* = 23, 62.2%), patient relevance (*N* = 14, 37.8%), data interpretation (*N* = 16, 43.2%), and flowchart reliability (*N* = 19, 51.4%).

Among the eight prognostic studies, the median number of patients was 143 (IQR = 66–217), and four (50.0%) studies showed a high risk of bias according to the QUIPS tool. Four (50.0%) did not provide information on the patients who dropped out, 2 (25.0%) did not adjust for important confounders and 4 (50.0%) had inadequate model strategy.

Among the twelve interventional trials, four RCTs, which included a median number of 56 patients (IQR = 29–111) were evaluated using RoB2 tool. Half of studies displayed a risk of bias for blinding in both the patients and the clinicians. Among the eight interventional non-randomized trials (median number of patients of 35 (IQR = 29–58)) evaluated using ROBINS-I tool, 6 (75.0%) were at risk of bias for not adjusting for important confounders, especially for post-intervention variables that could impact the effect of the intervention on outcome.

Out of 82 studies assessed with two evaluation tools to adequately address their design, 50 (61.0%) had consistent risk of bias evaluation, 10 (12.2%) were categorized as intermediate and high risk of bias, 17 (20.7%) were categorized as low and intermediate risk of bias, and 5 (6.1%) were categorized as low and high risk of bias (supplementary Figure 10).

## Discussion

In this comprehensive meta-research comprising 10,516 COVID-19-related medical articles that were screened, categorized and critically appraised, we have shown that the dynamic of publications since the start of the outbreak is mainly driven by publication without original data and differs across countries. We have also shown the topics addressed and that among the original articles, only few met the high scientific standards.

As highlighted in the results, the number of COVID-19-related medical articles is exponentially rising. A large number of case reports were published to share the medical experiences in the pandemic and may have served as an initial point for further studies. Moreover, many studies were published in the form of a research letter, which, while providing important data, is not a complete original study in its format and often lacks methodological information for quality assessment, affecting the application of the findings. Surprisingly, the peer-reviewed original articles accounted for only 10% of all COVID-19-related medical articles. Overall, the large number of publications without original data might be due to the readiness of health workers and researchers to control the spread of the virus and their willingness to share their experiences during the early stages of the pandemic.

We noted different publications trends based on the country of authors. As the earliest epidemic centre, China published many medical articles when the outbreak started, while in the western countries (e.g. UK and the US), many articles were released even before domestic COVID-19 infections were recognized. In Italy however, articles were starting to be published around the time when the first domestic COVID-19 infections were identified.

In addition, the findings consistently showed that the most represented topics were related to infectious disease, epidemiology, global health, and public health followed by studies related to health policy and medicine and society. Interestingly, simulation-based studies were also highly represented. Those studies were mainly conducted to predict the number of cases in different scenarios such as the adoption of different containment policies, demonstrating the concern and need for bringing the outbreak under control. Many medical specialties were represented, showing how physicians and researchers worldwide have communicated about their experiences and research about the COVID-19 [5–7], and the challenges most of healthcare workers are facing to fight the virus [32].

Of the 713 original articles we evaluated, the low proportion of high-quality articles was concerning, as less than 20% were at low risk of bias determined by validated tools. Interventional studies, which are critical for the discovery of effective drugs, were not only small in number but also at high risk of bias. The diagnostic and prognostic studies were of low quality and the results were consistent with a previous systematic review that, contrary to ours, focused on prediction models for prognosis and diagnosis of COVID-19 [33].

The assessment of research quality, and rigorous debate about the definition of quality, is a fundamental step in the advancement of scientific knowledge [21]. Many concerns have been raised by methodologists and researchers about the increased difficulty to converge towards scientific thoroughness in a pandemic, pressing time [9, 34]. This phenomenon of lowering of medical research standards has been previously highlighted [21]. For instance, several trials have been published, while using a small number of patients with scarce data [17]. Some articles were withdrawn, after having received widespread media attention [12, 13, 35]. Social medias may also play a role in the spread of misinformation, potentially relaying false of biased studies [9]. Overall, these phenomena underscore the need to strike a balance between the velocity of science, and the rigor of science [34].

This balance, however, may not be easily reachable. Our findings reveal that among the COVID-19-related medical articles, many were not peer-reviewed, interventional studies were often based on small case series data, and the risk of bias of original studies was overly high, illustrating the perception that standards tend to be revised downwards when it comes to a pandemic situation. In other words, one of the fundamental principles of medical science, that is, establishing associations with a high level of evidence, has been too often ignored, possibly justified by the necessity of sharing information for, in theory, a worldwide benefit [36]. The substantial risk to public health is that low quality scientific findings, which may be false, may draw valuable attention and resources away from valid scientific results [10, 21]. Researchers and medical professionals should remain aware of the noise surrounding the current medical literature, as science expediency may be higher in the present time.

In addition, in this context, the translation between medical research and clinical practice is essential. Many healthcare professionals importantly depends on what is currently published [37]. The frequent lack of reliability on articles quality and data may lead to inadequate decision-making and unfortunate consequences for the patient [10, 21]. In that sense, COVID-19 health researchers may have a more significant responsibility [16, 38]. The tremendous thirst for knowledge by the public and the perceived value of providing information quickly should not, however, influence the quality of research. More than ever, unproductive competition and opportunism should be avoided [20, 34] to publish relevant, rigorous, and reliable research [39]. Collaborations should be promoted and systematic reviews with regular updates are also urgently required for the health practitioners to gain a comprehensive understanding of the issues concerned [5, 33, 40, 41].

### Limitations

Several limitations should however be acknowledged. First, due to the exponential rise of COVID-19-related medical articles and hence the time constraints, references were not screened independently twice. This raises the issue of reproducibility in the assessment of each study’s risk of bias. However, our process of reviewer training and daily adjudication among reviewers may have minimized the risk of bias in the assessment of studies. Second, we did not assess the quality of research of preprints because of the very high number. Third, given the inclusion period, we could only assess a small number of interventional studies, although many are ongoing and will be published in the coming months.

## Conclusion

In conclusion, in this meta-research gathering both preprints and published COVID-19-related medical articles, we have presented the distribution of the different categories of medical publications, the dynamics of publications since the start of the outbreak, the variety of the topics addressed, and the poor quality of research of many peer-reviewed original articles. This study provides a deep understanding on COVID-19-related medical research and highlights medical topics of interest during the first phase of the pandemic. We acknowledge that, in this challenging time, emergency measures and rapid adaptation by healthcare workers, medical research is important and scientific communication should be promoted. Nevertheless, in light of this study, we urge healthcare researchers and practitioners to evaluate medical publications with appropriate skepticism despite the sense of urgency that the pandemic has generated, and to bear in mind that high standards of research are needed to make progress in controlling the pandemic: advances in medical science should be driven by compelling evidence, facilitating innovation and improvements in human health, especially during a pandemic, from the public health perspective to the individual care.

## Supplementary Information

**Additional file 1.**

## Data Availability

We stand ready to provide the references used in the present study to any researcher willing to reproduce the analysis. To do so, please contact the corresponding author.

## Abbreviations

COVID-19: Coronavirus disease 2019
RCT: Randomized controlled trial
UK: United Kingdom
US: United States
