# Supplementary material for: COVID-19-related medical research: a meta-research and critical appraisal
Source: BMC Med Res Methodol. 2021 Jan 4;21:1. doi: 10.1186/s12874-020-01190-w (PMC7780085; doi:10.1186/s12874-020-01190-w)
Supplement: Supplementary file 1 — Additional file 1. [file 12874_2020_1190_MOESM1_ESM.pdf]

Supplementary

Methods
1. PRISMA checklist
2. Search strategy
3. Risk of bias tool: description

Tables
Table 1. List of the topics addressed by all COVID-19-related medical articles
Table 2. MetaQAT tool for simulation studies
Table 3. AXIS tool for cross-sectional studies
Table 4. Checklist from M. H. Murad et al. for case series studies
Table 5. Newcastle-Ottawa Scale for cohort studies
Table 6. Newcastle-Ottawa Scale for case-control studies
Table 7. QUADAS-2 tool for diagnostic studies
Table 8. QUIPS tool for prognostic studies
Table 9. ROBINS-I tool for non-randomized interventional studies
Table 10. Cochrane Risk-of-Bias (RoB 2) tool for randomized controlled trials

Figures
Figure 1. Number of Covid-19-related medical articles of different types of publication
Figure 2. Dynamics of the accumulated number of Covid-19-related medical article since 2019-11-01: preprints vs peer-reviewed articles
Figure 3. Dynamics of the accumulated number of Covid-19-related medical article stratified by top 10 productive countries
Figure 4. Topic distribution of COVID-19-related, peer-reviewed medical articles
Figure 5. Topic distribution of COVID-19-related, preprint medical articles
Figure 6. Assessment of the quality of research in peer-reviewed original articles
Figure 7. Heatmap showing appraisal results of prognostic studies
Figure 8. Heatmap showing appraisal results of non-randomised interventional studies
Figure 9. Heatmap showing appraisal results of randomised controlled studies
Figure 10. Adequacy in the risk of bias for the 82 studies evaluated with two assessment tools.

METHODS

1. PRISMA Checklist

Section/topic	#	Checklist item	Reported on page #
**TITLE**			
Title	1	Identify the report as a systematic review, meta-analysis, or both.	1
**ABSTRACT**			
Structured summary	2	Provide a structured summary including, as applicable: background; objectives; data sources; study eligibility criteria, participants, and interventions; study appraisal and synthesis methods; results; limitations; conclusions and implications of key findings; systematic review registration number.	2
**INTRODUCTION**			
Rationale	3	Describe the rationale for the review in the context of what is already known.	3
Objectives	4	Provide an explicit statement of questions being addressed with reference to participants, interventions, comparisons, outcomes, and study design (PICOS).	3, 5
**METHODS**			
Protocol and registration	5	Indicate if a review protocol exists, if and where it can be accessed (e.g., Web address), and, if available, provide registration information including registration number.	5
Eligibility criteria	6	Specify study characteristics (e.g., PICOS, length of follow-up) and report characteristics (e.g., years considered, language, publication status) used as criteria for eligibility, giving rationale.	5
Information sources	7	Describe all information sources (e.g., databases with dates of coverage, contact with study authors to identify additional studies) in the search and date last searched.	5
Search	8	Present full electronic search strategy for at least one database, including any limits used, such that it could be repeated.	5
Study selection	9	State the process for selecting studies (i.e., screening, eligibility, included in systematic review, and, if applicable, included in the meta-analysis).	5, 6
Data collection process	10	Describe method of data extraction from reports (e.g., piloted forms, independently, in duplicate) and any processes for obtaining and confirming data from investigators.	5, 6
Data items	11	List and define all variables for which data were sought (e.g., PICOS, funding sources) and any assumptions and simplifications made.	6
Risk of bias in individual studies	12	Describe methods used for assessing risk of bias of individual studies (including specification of whether this was done at the study or outcome level), and how this information is to be used in any data synthesis.	7
Summary measures	13	State the principal summary measures (e.g., risk ratio, difference in means).	7
Synthesis of results	14	Describe the methods of handling data and combining results of studies, if done, including measures of consistency (e.g., I^2) for each meta-analysis.	7

Section/topic	#	Checklist item	Reported on page #
Risk of bias across studies	15	Specify any assessment of risk of bias that may affect the cumulative evidence (e.g., publication bias, selective reporting within studies).	7
Additional analyses	16	Describe methods of additional analyses (e.g., sensitivity or subgroup analyses, meta-regression), if done, indicating which were pre-specified.	Not applicable
**RESULTS**			
Study selection	17	Give numbers of studies screened, assessed for eligibility, and included in the review, with reasons for exclusions at each stage, ideally with a flow diagram.	8, 18
Study characteristics	18	For each study, present characteristics for which data were extracted (e.g., study size, PICOS, follow-up period) and provide the citations.	8, 20
Risk of bias within studies	19	Present data on risk of bias of each study and, if available, any outcome level assessment (see item 12).	9, 10
Results of individual studies	20	For all outcomes considered (benefits or harms), present, for each study: (a) simple summary data for each intervention group (b) effect estimates and confidence intervals, ideally with a forest plot.	Not applicable (number of studies too high)
Synthesis of results	21	Present results of each meta-analysis done, including confidence intervals and measures of consistency.	Not applicable
Risk of bias across studies	22	Present results of any assessment of risk of bias across studies (see Item 15).	9, 10
Additional analysis	23	Give results of additional analyses, if done (e.g., sensitivity or subgroup analyses, meta-regression [see Item 16]).	8, 9, 10, 19
**DISCUSSION**			
Summary of evidence	24	Summarize the main findings including the strength of evidence for each main outcome; consider their relevance to key groups (e.g., healthcare providers, users, and policy makers).	12, 13
Limitations	25	Discuss limitations at study and outcome level (e.g., risk of bias), and at review-level (e.g., incomplete retrieval of identified research, reporting bias).	14
Conclusions	26	Provide a general interpretation of the results in the context of other evidence, and implications for future research.	14, 15
**FUNDING**			
Funding	27	Describe sources of funding for the systematic review and other support (e.g., supply of data); role of funders for the systematic review.	15

2. Search strategy

Pubmed and the Cochrane COVID-19 study register were used to identify peer-reviewed articles. BioRxiv, medRxiv and arXiv data sources were used to identify preprints. We defined different search strategies according to the data sources. The protocol of the study is available at: **https://osf.io/5zjyx/**. The publications related to COVID-19 have been identified by title and abstract.

Search string in PubMed

2019-nCoV OR wuhan coronavirus OR China coronavirus OR novel coronavirus OR SARS-CoV-2 OR "severe acute respiratory syndrome coronavirus 2" OR COVID-19 OR coronavirus disease 2019 OR Novel Coronavirus Pneumonia OR "COVID-19 vaccine" [Supplementary Concept] OR "COVID-19 diagnostic testing" [Supplementary Concept] OR "severe acute respiratory syndrome coronavirus 2" [Supplementary Concept] OR "COVID-19" [Supplementary Concept] OR "spike glycoprotein, COVID-19 virus" [Supplementary Concept] OR "COVID-19 serotherapy" [Supplementary Concept] OR "COVID-19 drug treatment" [Supplementary Concept]

Search string in Cochrane Covid-19 study register

The register provides a list of research articles on Covid-19 with the following link (https://covid-19.cochrane.org/)

Search string in bioRxiv, medRxiv, arXiv

The bioRxiv and medRxiv preprint servers provide a list of research articles on COVID-19 with the following link (https://connect.medrxiv.org/relate/content/181 or https://connect.biorxiv.org/relate/content/181). The search term in arXiv preprint server is (by default in the website https://arxiv.org/): title=COVID-19; OR abstract=SARS-CoV-2; OR abstract=COVID-19; OR title=SARS-CoV-2; OR title=coronavirus; OR abstract=coronavirus.

3. Risk of bias tools: description

To critically appraise the COVID-19 original articles, we used several tools to address all study designs. The table below presents each tool and how they were ultimately categorized to depict the overall risk of bias for each study.

Study design	Quality assessment tool	Description	Risk of bias		
			High	Intermediate	Low
**Simulation studies**	MetaQAT[1]	The MetaQAT framework consists of four domains of assessment: relevancy, reliability, validity and applicability.	If findings bias item has not been adequately addressed, or if two or more items have not been adequately addressed	If exactly one item has not been adequately addressed	If all items have been adequately addressed
**Cross-sectional studies**	AXIS[2]	The AXIS tool consists of 20 components: seven of them are related to quality of reporting, seven of them to study design quality and six are related to the possible introduction of biases in the study	If study design or population representativeness or selection process or data description or parameters measurements have not been adequately addressed, or if five or more items have not been adequately addressed	If exactly three or four items have not been adequately addressed, and are not among those described in the high-risk group	If three or less items have not been adequately addressed, and are not among those described in the high-risk group
**Case series**	Checklist from M. H. Murad et al.[3]	This checklist is based on the domains of selection, ascertainment, causality and reporting	If patient representativeness or follow-up duration or explaining observation ruled out or data description have not been adequately addressed, or if two items or more have not been adequately addressed	If exactly one item has not been adequately addressed, and is not among those described in the high-risk group	If all items have been adequately addressed
**Cohort studies**	NOS for cohort studies[4]	Based on the NOS tool, the study is appraised according to three broad perspectives: the selection of the study groups, the comparability of the groups and the ascertainment of either the exposure or outcome of interest for case-control or cohort studies respectively	If the overall score is equal or lower than 6	If the overall score is equal to 7 or 8	If the overall score is equal to 9
**Case-control studies**	NOS for case-control studies[4]		If the overall score is equal or lower than 6	If the overall score is equal to 7 or 8	If the overall score is equal to 9
**Diagnostic studies**	QUADAS-2[5]	This tool comprises 4 domains: patient selection, index test, reference standard, and flow and timing	If patient flow bias or selection bias or interpretation bias or patient relevance have not been adequately addressed, or if two items or more have not been adequately addressed	If exactly one item has not been adequately addressed, and is not among those described in the high-risk group	If all items have been adequately addressed
**Prognostic studies**	QUIPS[6]	This tool comprises 6 domains: study participation, study attrition, prognostic factor measurement, outcome measurement, study confounding, and statistical analysis and reporting	If at least one domain is categorized as high risk of bias	If at least two domains are categorized as intermediate risk of bias, and others are categorized as low risk of bias	If all domains are categorized as low risk of bias, or if a maximum of one item is categorized as

					intermediate risk of bias and all others as low risk of bias
**Non-randomized interventional studies**	ROBINS-I[7]	This tool addresses seven domains through which bias might be introduced into a non-randomised interventional study. The two first domains cover issues before the start of the interventions: bias due to confounding and bias in selection of participants into the study. The third domain addresses bias in classification of the interventions themselves. The other four domains address issues after the start of interventions: biases due to deviations from intended interventions, bias due to missing data, bias in measurement of outcomes and bias in selection of the reported result	The study is judged to be at serious or critical risk of bias in at least one domain	The study is judged to be at low or moderate risk of bias for all domains, with at least one domain being at moderate risk of bias	The study is judged to be at low risk of bias for all domains
**Randomized controlled trials**	Cochrane RoB 2[8]	RoB 2 is structured into five bias domains, which were selected to address all important mechanisms by which bias can be introduced into the results of a trial, based on a combination of empirical evidence and theoretical considerations : bias arising from the randomisation process, bias due to deviations from intended interventions, bias due to missing outcome data, bias in measurement of the outcome and bias in selection of the reported result.	If at least one domain is at high risk of bias, or if the study is judged to have some concerns for multiple domains	The study is judged to raise some concerns in at least one domain for this result, but not to be at high risk of bias for any domain	The study is judged to be at low risk of bias for all domains for this result

TABLES

Table 1. List of the topics addressed by all COVID-19-related medical articles
During the screening, reviewers categorized the papers into up to three topics. The following initial list of topics was defined before the review start: Health policy, Medicine and society, Intensive care, Epidemiology, Infectious disease, Prognosis, Diagnosis, Telemedicine, Cardiology, Internal medicine, Oncology, Psychiatry, Nephrology, Treatment, Vaccine, Global health, Public health, Etiology, Virology, Prevention, Basic science, Psychology, Dermatology, Neurology, Ophthalmology, Surgery, Radiology, Pediatrics, Genetics, Geriatrics and Rheumatology. This list was then continuously supplemented with new topic, if appropriate. This table shows the final subcategories after the screening (N=200 subcategories), further reduced to 45 main categories after discussion and consensus with the consortium.

Main categories	Subcategories
**Infectious diseases**<br><br>*Investigating the disease, the origin, how it spreads, characterizing the populations at risk, and discussing diagnosis, management and treatment of COVID-19 patients*	• Infectiology<br>• Pandemics<br>• Controlling the infection source<br>• Exported infections<br>• Viral infections<br>• Zoonosis<br>• Infectious disease
**Virology**<br><br>*Studying the virus in itself*	• Origin of virus<br>• Phylogeny<br>• Virology
**Disease transmission**<br><br>*Investigating the mode of transmission, the rate of transmission, and proposing guidance/warnings accordingly*	• Transmission
**Epidemiology**<br><br>*Investigating the incidence, prevalence, risk factors and associated health outcomes of COVID-19 patients*	• Prognosis<br>• Epidemiology<br>• Risk factors
**Simulation**<br><br>*Used advanced statistical approaches to simulate, estimate or predict outcomes linked to the virus*	• Simulation
**Public health**<br><br>*Describing how countries locally responded to disease spread, which health policies were adopted, the strategies for prevention, containment and surveillance of the virus*	• Health policy<br>• Environment<br>• Surveillance<br>• Survey<br>• Protection of at risk-population<br>• Public health

**Disease control**	• Disease control
**Prevention**	• Prevention
**Global health**<br><br>*Presenting how the health systems worldwide adapt to the pandemics, and sharing experiences, practices and guidelines that may benefit internationally*	• Health care delivery<br>• Patient management<br>• World Health organization<br>• Health organization<br>• Medical ethics<br>• Practice management<br>• Poor level population<br>• Global health<br>• Health research<br>• Scientific communication<br>• Humanity<br>• Nursing
**Laboratory Medicine**	• Laboratory Medicine
**Therapeutics, Drugs and Medicines**	• Pharmacology<br>• Pharmacy<br>• Immunosuppressive therapy<br>• Immunotherapy<br>• Faecal microbiota transplantation<br>• Clinical trial<br>• Adverse effects<br>• Chemical drugs<br>• Multidisciplinary therapy<br>• Non-pharmacological Treatment<br>• Traditional chinese medicine<br>• Non-medicine therapy
**Vaccine**	• Vaccine
**Diagnostics**	• Diagnostic test<br>• Screening<br>• Serological test<br>• Serology<br>• Symptoms<br>• Diagnostic biomarker<br>• Diagnostics
**Medicine and society**<br><br>*Depicting which information and tools citizens have in the pandemic situation (telemedicine, online education)*	• Media<br>• Telemedicine<br>• Art and medicine<br>• General practice<br>• Growth and development<br>• Online education<br>• Infodemiology<br>• Knowledge map<br>• Commerce<br>• Communication<br>• Device<br>• Education<br>• Exercise
**Methodology**<br><br>*Practice*	• Study design<br>• Code of practice<br>• Consensus<br>• Guidelines<br>• Methodology
**Radiology**	• Imaging<br>• Ultrasonography<br>• Radiology
**Genetics**	• Molecular<br>• Genomics

	• Molecular medicine<br>• Genetics
**Immunology**	• Allergy<br>• Immune deficiency<br>• Immunity<br>• Immunohematology<br>• Immunology
**Intensive care**	• Emergency medicine<br>• Burnt victims<br>• Intensive care<br>• Critical care
**Pediatrics**	• Pediatrics
**Health workers**	• Health workers
**Mental health**	• Mental health<br>• Behavioural science<br>• Sleep disorders<br>• Psychiatry<br>• Psychology
**Artificial intelligence**	• Machine learning<br>• Bioinformatics<br>• Computational biology<br>• AI
**Basic science**<br>*Translational and biological medicine*	• Biology<br>• Biochemistry<br>• Pathogenesis<br>• Biophysics<br>• Epigenetics<br>• Microbiology<br>• Pathophysiology<br>• Protein structure<br>• Physiology<br>• Cellular biology<br>• Animals<br>• Animal welfare<br>• Mechanisms<br>• Nanosciences<br>• Scientific research<br>• Transmission medium
**Cardiology**	• Hypertension<br>• Myocardial damage<br>• Cardiology<br>• Cardiovascular health
**Hematology**	• Hematology<br>• Blood donation<br>• Blood transfusion<br>• Haemostasis<br>• Transfusion<br>• Coagulation disorders<br>• Plasma
**Oncology**	• Oncology<br>• Cancer<br>• Breast Cancer

**Hepato-gastroenterology**	• Gastroenterology<br>• Bowel diseases<br>• Endoscopy<br>• Hepatology<br>• Liver
**Endocrinology**	• Endocrinology<br>• Diabetes<br>• Hormone<br>• Nutrition<br>• Obesity
**Pathology**	• Pathology<br>• Histology<br>• Forensic pathology<br>• Autopsy
**Obstetrics and Gynaecology**	• Obstetrics<br>• Gynaecology<br>• Reproductive medicine<br>• Pregnancy
**Internal medicine**	• Primary care<br>• Physical and rehabilitation medicine
**Nephrology**	• Nephrology
**Data sharing**	• Data storage<br>• Data access
**Transplantation**	• Cell transplantation<br>• Transplantation<br>• Kidney transplantation
**Others**	• Astronomy<br>• Geography<br>• Journal policies<br>• Laboratory medicine<br>• Taxonomy<br>• Toxicology<br>• Podiatric<br>• Nuclear medicine<br>• Electronic health records<br>• AIDS<br>• Researching tools<br>• Nomenclature<br>• Terminology<br>• Veterinary<br>• Economy<br>• Fecal transmission
**Pneumology**	• Interventional pulmonology<br>• Smoking
**Dermatology**	• Dermatology
**Anaesthesia**	• Anaesthesia

**Geriatrics**	• Aging<br>• Geriatrics
**Palliative medicine**	• Palliative care
**Neurology**	• Migraine<br>• Neuroscience<br>• Spinal disease
**Ophthalmology**	• Ophthalmology
**Surgery**	• Orthopaedics<br>• Traumatology<br>• Urology<br>• Digestive surgery
**Otorhinolaryngology**	• Stomatology<br>• Otolaryngologists<br>• Otolaryngology<br>• Oral disease<br>• Oral medicine<br>• Dentistry

Table 2. MetaQAT for simulation studies

MetaQAT for simulation studies	
**Relevancy**	
**Items**	**Answers**
Is the study presented clearly?	A) Yes<br>B) No<br>C) Unclear<br>D) Not appropriate
**Reliability**	
1) Is the study presented clearly?	A) Yes<br>B) No<br>C) Unclear<br>D) Not appropriate
2) Are the research methodology and results clearly described?	A) Yes<br>B) No<br>C) Unclear<br>D) Not appropriate
3) Are ethics procedures described?	A) Yes<br>B) No<br>C) Unclear<br>D) Not appropriate
**Validity**	
1) Is the study methodology appropriate for the scope of research?	A) Yes<br>B) No<br>C) Unclear<br>D) Not appropriate
2) Is the research methodology free from bias?	A) Yes<br>B) No<br>C) Unclear<br>D) Not appropriate
3) Are the authors' conclusions explicit and transparent?	A) Yes<br>B) No<br>C) Unclear<br>D) Not appropriate
4) Can I be confident about the findings?	A) Yes<br>B) No<br>C) Unclear<br>D) Not appropriate
**Applicability**	
How can the results be applied within the scope public health?	A) Yes<br>B) No<br>C) Unclear<br>D) Not appropriate

Table 3. AXIS for cross-sectional studies

General indication: Question 5, 6 and 7 don't apply to census studies in theory.

AXIS for cross-sectional studies critical appraisal		
**Introduction**		
**Items**	**Answers**	**Comment**
1) Were the aims/objectives of the study clear?	A) Yes<br>B) No<br>C) Don't know	
**Methods**		
2) Was the study design appropriate for the stated aim(s)?	A) Yes<br>B) No<br>C) Don't know	
3) Was the sample size justified?	A) Yes<br>B) No<br>C) Don't know	
4) Was the target/reference population clearly defined? (Is it clear who the research was about?)	A) Yes<br>B) No<br>C) Don't know	
5) Was the sample frame taken from an appropriate population base so that it closely represented the target/reference population under investigation?	A) Yes<br>B) No<br>C) Don't know	
6) Was the selection process likely to select subjects/participants that were representative of the target/reference population under investigation?	A) Yes<br>B) No<br>C) Don't know	
7) Were measures undertaken to address and categorize non-responders?	A) Yes<br>B) No<br>C) Don't know	
8) Were the risk factor and outcome variables measured appropriate to the aims of the study?	A) Yes<br>B) No<br>C) Don't know	
9) Were the risk factor and outcome variables measured correctly using instruments that had been trialed, piloted or published previously?	A) Yes<br>B) No<br>C) Don't know	
10) Is it clear what was used to determined statistical significance and /or precision estimates? (e.g. P-values, confidence intervals)	A) Yes<br>B) No<br>C) Don't know	
11) Were the methods (including statistical methods) sufficiently described to enable them to be repeated?	A) Yes<br>B) No<br>C) Don't know	
**Results**		
12) Were the basic data adequately described?	A) Yes<br>B) No<br>C) Don't know	
13) Dose the response rate raise concerns about non-response bias?	A) Yes<br>B) No<br>C) Don't know	

14) If appropriate, was information about non-responders described?	A) Yes<br>B) No<br>C) Don't know	
15) Were the results internally consistent?	A) Yes<br>B) No<br>C) Don't know	
16) Were the results presented for all the analyses described in the methods?	A) Yes<br>B) No<br>C) Don't know	
**Discussion**		
17) Were the authors' discussions and conclusions justified by the results?	A) Yes<br>B) No<br>C) Don't know	
18) Were the limitations of the study discussed?	A) Yes<br>B) No<br>C) Don't know	
**Other**		
19) Were there any funding source or conflicts of interest that may affect the authors' interpretation of the results?	A) Yes<br>B) No<br>C) Don't know	
20) Was ethical approval or consent of participants attained?	A) Yes<br>B) No<br>C) Don't know	

Table 4. Checklist from M. H. Murad et al. for case series studies

General indications: A study can be evaluated by answering eight items with leading explanatory questions. The answer can be "Yes", "No", or "Not applicable". Questions 4, 5 and 6 are mostly relevant to cases of adverse drug events.

Checklist by M. H. Murad et al.	
**Selection**	
**Items**	**Answers**
1. Does the patient(s) represent(s) the whole experience of the investigator (centre) or is the selection method unclear to the extent that other patients with similar presentation may not have been reported?	Yes/ No/ Not applicable
**Ascertainment**	
2. Was the exposure adequately ascertained?	Yes/ No/ Not applicable
3. Was the outcome adequately ascertained?	Yes/ No/ Not applicable
**Causality**	
4. Were other alternative causes that may explain the observation ruled out?	Yes/ No/ Not applicable
5. Was there a challenge/rechallenge phenomenon?	Yes/ No/ Not applicable
6. Was there a dose–response effect?	Yes/ No/ Not applicable
7. Was follow-up long enough for outcomes to occur?	Yes/ No/ Not applicable
**Reporting**	
8. Is the case(s) described with sufficient details to allow other investigators to replicate the research or to allow practitioners make inferences related to their own practice?	Yes/ No/ Not applicable

Table 5. Newcastle-Ottawa scale for cohort studies

<u>General indications</u>: A study can be awarded a maximum of one point for each numbered item within the Selection and Outcome categories. A maximum of two points can be given for Comparability. Columns "Sum" = sum of points of each item: Selection (0 to 4), Comparability (0 to 2) and Outcome (0 to 3).

New Ottawa Scale for risk of bias in cohort studies	
**Selection**	
**Items**	**Answers**
1) Representativeness of the exposed cohort	A) Truly representative of the average...(describe) in the community (1point)<br>B) Somewhat representative of the average ... in the community (1 point)<br>C) Selected group of users eg nurses, volunteers<br>D) No description of the derivation of the cohort
2) Selection of the non-exposed cohort	A) Drawn from the same community as the exposed cohort (1 point)<br>B) Drawn from a different source<br>C) No description of the derivation of the non-exposed cohort
3) Ascertainment of exposure	A) Secure record (eg surgical records) (1 point)<br>B) Structured interview (1 point)<br>C) Written self-report<br>D) No description
4) Demonstration that outcome of interest was not present at start of study	A) Yes (1 point)<br>B) No
**Comparability**	
Comparability of cohorts on the basis of the design or analysis	A) Study controls for ... (select the most important factor) (1 point)<br>B) Study controls for any additional factor (This criteria could be modified to indicate specific control for a second important factor.) (1 point)
**Outcome**	
1) Assessment of outcome	A) Independent blind assessment (1 point)<br>B) Record linkage (1 point)<br>C) Self-report<br>D) No description
2) Was follow-up long enough for outcomes to occur	A) Yes (select an adequate follow up period for outcome of interest) (1 point)<br>B) No
3) Adequacy of follow up of cohorts	A) Complete follow up - all subjects accounted for (1 point)<br>B) Subjects lost to follow up unlikely to introduce bias - small number lost < (select an adequate %) follow up, or description provided of those lost) (1 point)<br>C) Follow up rate < ____% (select an adequate %) and no description of those lost<br>D) No statement

Table 6. Newcastle-Ottawa scale for case-control studies

General indications: A study can be awarded a maximum of one point for each numbered item within the Selection and Exposure categories. A maximum of two points can be given for Comparability. Columns "Sum" = sum of points of each item: selection (0 to 4), Comparability (0 to 2) and Exposure (0 to 3).

**New Ottawa Scale for risk of bias in case-control studies**	
**Selection**	
**Items**	**Answers**
1) Is the case definition adequate?	A) Yes, with independent validation (1 point) (> 1 person/record/time/process to extract information, or reference to primary record source such as x-rays or medical/hospital records).<br>B) Yes, eg record linkage or based on self-reports<br>C) No description
2) Representativeness of the cases	A) Consecutive or obviously representative series of cases (1 point)<br>B) Potential for selection biases or not stated
3) Selection of Controls	A) Community controls (1 point)<br>B) Hospital controls<br>C) No description
4) Definition of Controls	A) No history of disease (endpoint) (1 point)<br>B) No description of source
**Comparability**	
Comparability of cases and controls on the basis of the design or analysis	A) Study controls for the most important factor (eg PCR+, serology…) (1 point)<br>B) Study controls for any additional factor (this criterion could be modified to indicate specific control for a second important factor). (1 point)
**Exposure**	
1) Ascertainment of exposure (1 point if A or B)	A) Secure record (eg surgical records)<br>B) Structured interview where blind to case/control status<br>C) Not Blinded interview not blinded to case/control status<br>D) Written self-report or medical record only<br>E) No description
2) Same method of ascertainment for cases and controls	A) Yes (1 point)<br>B) No
3) Non-Response rate	A) Same rate for both groups (1 point)<br>B) Non-respondents described<br>C) Rate different and no designation

Table 7. QUADAS-2 tool for diagnostic studies

QUADAS-2 for risk of bias in diagnostic studies	
**Patient selection**	
**Items**	**Answers**
1) Was a consecutive or random sample of patients enrolled?	A) Yes<br>B) No<br>C) Unclear
2) Was a case-control design avoided?	A) Yes<br>B) No<br>C) Unclear
3) Did the study avoid inappropriate exclusions?	A) Yes<br>B) No<br>C) Unclear
4) Could the selection of patients have introduced bias?	A) Low<br>B) High<br>C) Unclear
5) Is there concern that the included patients do not match the review question?	A) Low<br>B) High<br>C) Unclear
**Index test**	
6) Were the index test results interpreted without knowledge of the results of the reference standard?	A) Yes<br>B) No<br>C) Unclear
2) If a threshold was used, was it pre-specified?	A) Yes<br>B) No<br>C) Unclear
3) Could the conduct or interpretation of the index test have introduced bias?	A) Low<br>B) High<br>C) Unclear
4) Is there concern that the index test, its conduct, or interpretation differ from the review question?	A) Low<br>B) High<br>C) Unclear
**Reference standard**	
1) Is the reference standard likely to correctly classify the target condition?	A) Yes<br>B) No<br>C) Unclear
2) Were the reference standard results interpreted without knowledge of the results of the index test?	A) Yes<br>B) No<br>C) Unclear
3) Could the reference standard, its conduct, or its interpretation have introduced bias?	A) Low<br>B) High<br>C) Unclear
Is there concern that the target condition as defined by the reference standard does not match the review question?	A) Low<br>B) High<br>C) Unclear
**Flow and timing**	
Was there an appropriate interval between index test(s) and reference standard?	A) Yes<br>B) No<br>C) Unclear

Did all patients receive a reference standard?	A) Yes<br>B) No<br>C) Unclear
Did patients receive the same reference standard?	A) Yes<br>B) No<br>C) Unclear
Were all patients included in the analysis?	A) Yes<br>B) No<br>C) Unclear
Could the patient flow have introduced bias?	A) Low<br>B) High<br>C) Unclear

Table 8. QUIPS for prognostic studies

Biases	Issues to consider for judging overall rating of "Risk of bias"	Judgement
**1. Study Participation**	**Goal: To judge the risk of selection bias**	**YES/NO**
Source of target population	The source population or population of interest is adequately described for key characteristics	
Method used to identify problem	The sampling frame and recruitment are adequately described, possibly including methods to identify the sample, place of recruitment, and period of recruitment	
Inclusion and exclusion criteria	Inclusion and exclusion criteria are adequately described	
Adequate study participation	There is adequate participation in the study by eligible individuals	
Baseline characteristics	The baseline study sample is adequately described for key characteristics	
**Summary Study Participatio n**	**The study sample represents the population of interest on key characteristics, sufficient to limit potential bias of the observed relationship between the prognostic factor and outcome**	
OVERALL RISK OF BIAS (low/intermediate/high)		
**2. Study Attrition**	**Goal: To just the risk of attrition bias**	
Proportion of baseline sample available for analysis	Response rate is adequate and is > 80%	
Attempts to collect information on participants who dropped out	Attempts to collect information on participants who dropped out of the study are described	
Reasons and potential impact of subjects lost to follow up	Reasons for loss to follow up are described	
Outcome and prognostic factor information on those lost to follow up	Participants lost to follow up are adequately described for key characteristics	
	There are no important differences between key characteristics and outcomes in participants who completed the study and those who did not	

**Summary Study Attrition**	**Loss to follow-up is not associated with key characteristics sufficient to limit potential bias to the observed relationship between the prognostic factor and the outcome**	
OVERALL RISK OF BIAS (low/intermediate/high)		
**3. Prognostic Factor Measurement**	**Goal: To judge the risk of measurement bias related to how the prognostic factor was measured**	
Definition of the PF	A clear definition or description of the prognostic factors is provided	
Valid and reliable measurement of PF	Method of prognostic factor measurement is adequately valid and reliable to limit misclassification bias	
	The prognostic factors measured are blinded for outcome measure	
	Continuous variables are reported or appropriate cut-offs are used	
Method and setting of PF measurement	The method and setting of measurement of PF is the same for all study participants	
Proportion of data on PF available for analysis	More than 80% of the study sample has completed data for PF variable	
Method used for missing data	Appropriate methods of imputation are used for missing 'PF' data	
**PF Measurement Summary**	**PF is adequately measured in study participants to sufficiently limit potential bias**	
OVERALL RISK OF BIAS (low/intermediate/high)		
**4. Outcome Measurement**	**Goal: To judge the risk of bias related to the measurement of outcome**	
Definition of the Outcome	A clear definition of the Outcome is provided	
Valid and reliable measurement of Outcome	The method of outcome measurement used in valid and reliable to limit misclassification bias	
Method and setting of Outcome Measurement	The method and setting of outcome measurement is the same for all study participants	
**Outcome Measurement Summary**	**Outcome of interest is adequately measured in study participants to sufficiently limit potential bias**	
OVERALL RISK OF BIAS (low/intermediate/high)		
**5. Study Confounding**	**Goal: To judge the risk of bias due to confounding**	

Important Confounders measured	All important confounders are measured	
Definition of the confounding factor	Clear definitions of the important confounders measured are provided	
Method and setting of Confounding Measurement	The method and setting of confounding measurement are the same for all study participants	
Appropriate accounting for confounding	Important potential confounders are accounted for in the study design	
	Important potential confounders are accounted for in the analysis	
**Study Confounding Summary**	**Important potential confounders are appropriately accounted for, limiting potential bias with respect to the relationship between PF and outcome**	
OVERALL RISK OF BIAS (low/intermediate/high)		
**6. Statistical Analysis and Reporting**	**Goal: To judge the risk of bias related to the statistical analysis and presentation of results**	
Presentation of analytical strategy	There is sufficient presentation of data to assess the adequacy of the analysis	
Model development strategy	The strategy for model building is appropriate and is based on a conceptual framework or model.	
	The selected statistical model is adequate for the design of the study	
Reporting of results	There is a description of the association of the prognostic factor and the outcome, including information about the statistical significance	
	Continuous variables are reported or cut-off points are used	
	There is no selective reporting of results	
**Statistical Analysis and Reporting Summary**	**The statistical analysis is appropriate for the design of the study, limiting potential for presentation of invalid or spurious results**	
OVERALL RISK OF BIAS (low/intermediate/high)		

Table 9. Risk Of Bias In Non-randomized Studies of Interventions (ROBINS-I) tool for non-randomized interventional studies

General indications: ROBINS-I tool is concerned with evaluating the risk of bias (RoB) in the results of non-randomized studies (NRSIs) that compare the health effects of two or more interventions. The ROBINS-I tool covers seven domains through which bias might be introduced into a NRSI. There are several signalling questions within each domain of bias. The response options for the signalling questions are: (1) Yes (Y); (2) Probably yes (PY); (3) Probably no (PN); (4) No (N); and (5) No information (NI). Domain-level judgements about risk of bias are made and an overall judgement about risk of bias is reached.

**Risk Of Bias In Non-randomized Studies of Interventions (ROBINS-I)**	
**Bias due to confounding**	
**Items**	**Answers**
1.1 Is there potential for confounding of the effect of intervention in this study? If N/PN => low risk	Y / PY / PN / N
1.2. If Y/PY to 1.1. : Was the analysis based on splitting participant's follow-up time according to intervention received? If N/PN => go to 1.4 to 1.6 ; if Y/PY => go to 1.3.	NA / Y / PY / PN / N / NI
1.3. Were intervention discontinuations or switches likely to be related to factors that are prognostic for the outcome? If N/PN=> go to 1.4 to 1.6, if Y/PY, go to 1.7-1.8	NA / Y / PY / PN / N / NI
1.4 Did the authors use an appropriate analysis method that controlled for all the important confounding domains?	NA / Y / PY / PN / N / NI
1.5. If Y/PY to 1.4 : Were confounding domains that were controlled for measured validly and reliably by the variables available in this study?	NA / Y / PY / PN / N / NI
1.6. Did the authors control for any post-intervention variables that could have been affected by the intervention?	NA / Y / PY / PN / N / NI
1.7. Did the authors use an appropriate analysis method that adjusted for all the important confounding domains and for time-varying confounding?	NA / Y / PY / PN / N / NI
1.8. If Y/PY to 1.7 : Were confounding domains that were adjusted for measured validly and reliably by the variables available in this study?	NA / Y / PY / PN / N / NI
Risk of Bias judgement	Low / Moderate / Serious / Critical / NI
**Bias in selection of participants into the study**	
2.1. Was selection of participants into the study (or into the analysis) based on participant characteristics observed after the start of intervention? If N/PN => go to 2.4	Y / PY / PN / N / NI

2.2 : if Y/PY to 2.1 : Were the post-intervention variables that influenced selection likely to be associated with intervention?	NA / Y / PY / PN / N / NI
2.3 if Y/PY to 2.2 : Were the post-intervention variables that influenced selection likely to be influenced by the outcome or a cause of the outcome?	NA / Y / PY / PN / N / NI
2.4.Do start of follow up and start of intervention coincide for most participants?	Y / PY / PN / N / NI
2.5. If Y/PY to 2.2 and 2.3, or N/PN to 2.4 : Were adjustment techniques used that are likely to correct for the presence of selection biases?	NA / Y / PY / PN / N / NI
Risk of Bias Judgement	Low / Moderate / Serious / Critical / NI
**Bias in classification of interventions**	
3.1 Were interventions groups clearly defined?	Y / PY / PN / N / NI
3.2 Was the information used to define intervention groups recorded at the start of the intervention?	Y / PY / PN / N / NI
3.3 Could classification of intervention status have been affected by knowledge of the outcome or risk of the outcome?	Y / PY / PN / N / NI
Risk of Bias Judgement	Low / Moderate / Serious / Critical / NI
**Bias due to deviations from intended interventions**	
4.1 Were there deviations from the intended intervention beyond what would be expected in usual practice?	Y / PY / PN / N / NI
4.2 If Y/PY to 4.1 : Were these deviations from intended intervention unbalanced between groups and likely to have affected the outcome?	NA / Y / PY / PN / N / NI
4.3 Were important co-interventions balanced across intervention groups?	Y / PY / PN / N / NI
4.4. Was the intervention implemented successfully for most participants?	Y / PY / PN / N / NI
4.5. Did study participants adhere to the assigned intervention regimen?	Y / PY / PN / N / NI

4.6. If N/PN to 4.3, 4.4 or 4.5 : Was an appropriate analysis used to estimate the effect of starting and adhering to the intervention?	NA / Y / PY / PN / N / NI
Risk of Bias Judgement	Low / Moderate / Serious / Critical / NI
**Bias due to missing data**	
5.1 Were outcome data available for all, or nearly all, participants?	Y / PY / PN / N / NI
5.2 Were participants excluded due to missing data on intervention status?	Y / PY / PN / N / NI
5.3 Were participants excluded due to missing data on other variables needed for the analysis?	Y / PY / PN / N / NI
5.4 If PN/N to 5.1, or Y/PY to 5.2 or 5.3 : Are the proportion of participants and reasons for missing data similar across interventions?	NA / Y / PY / PN / N / NI
5.5 If PN/N to 5.1, or Y/PY to 5.2 or 5.3 : Is there evidence that results were robust to the presenceof missing data?	NA / Y / PY / PN / N / NI
Risk of Bias Judgement	Low / Moderate / Serious / Critical / NI
**Bias in measurement of outcomes**	
6.1 : Could the outcome measure have been influenced by knowledge of the intervention received?	Y / PY / PN / N / NI
6.2. Were outcome assessors aware of the intervention received by study participants?	Y / PY / PN / N / NI
6.3. Were the methods of outcome assessment comparable across intervention groups?	Y / PY / PN / N / NI
6.4 : Were any systematic errors in measurement of the outcome related to intervention received?	Y / PY / PN / N / NI
Risk of Bias Judgement	Low / Moderate / Serious / Critical / NI
**Bias in selection of the reported result : Is the reported effect estimate likely to be selected, on the basis of the results, from…**	
7.1. … multiple outcome measurements within the outcome domain?	Y / PY / PN / N / NI
7.2. … Multiple analyses of the intervention-outcome relationship?	Y / PY / PN / N / NI
7.3. … different subgroups?	Y / PY / PN / N / NI

Risk of Bias Judgement	Low / Moderate / Serious / Critical / NI

Table 10. Cochrane Risk-of-Bias (RoB 2) tool for randomized controlled trials

General indications:
RoB 2 is structured into five bias domains: bias arising from the randomization process, bias due to deviations from intended interventions (effect of assignment to intervention and effect of adhering to intervention), bias due to missing outcome data, bias in measurement of the outcome and bias in selection of the reported result. Answers for each item in each domain were: 'Yes', 'Probably Yes', 'Probably No', 'No' and 'No information'. Answers for risk-of-bias judgement in each domain were: 'Low', 'High', 'Some concerns'. Answers for overall risk-of-bias judgement were: 'Low', 'High', 'Some concerns'.

**RoB2 tool for risk of bias in RCT**	
**Domain 1: Risk of bias arising from the randomization process**	
**Items**	**Answers**
**1) Was the allocation sequence random?**	'Yes' if a random component was used in the sequence generation process. Examples include computer-generated random numbers; reference to a random number table; coin tossing; shuffling cards or envelopes; throwing dice; or drawing lots. Minimization is generally implemented with a random element (at least when the scores are equal), so an allocation sequence that is generated using minimization should generally be considered to be random.<br>'No' if no random element was used in generating the allocation sequence or the sequence is predictable. Examples include alternation; methods based on dates (of birth or admission); patient record numbers; allocation decisions made by clinicians or participants; allocation based on the availability of the intervention; or any other systematic or haphazard method.<br>'No information' if the only information about randomization methods is a statement that the study is randomized.<br>In some situations a judgement may be made to answer 'Probably no' or 'Probably yes'. For example, in the context of a large trial run by an experienced clinical trials unit, absence of specific information about generation of the randomization sequence, in a paper published in a journal with rigorously enforced word count limits, is likely to result in a response of 'Probably yes' rather than 'No information'. Alternatively, if other (contemporary) trials by the same investigator team have clearly used non-random sequences, it might be reasonable to assume that the current study was done using similar methods.
**2) Was the allocation sequence concealed until participants were enrolled and assigned to interventions?**	'Yes' if the trial used any form of remote or centrally administered method to allocate interventions to participants, where the process of allocation is controlled by an external unit or organization, independent of the enrolment personnel (e.g. independent central pharmacy, telephone or internet-based randomization service providers). Answer 'Yes' if envelopes or drug containers were used appropriately. Envelopes should be opaque, sequentially numbered, sealed with a tamper-proof seal and opened only after the envelope has been irreversibly assigned to the participant. Drug containers should be sequentially numbered and of identical appearance, and dispensed or administered only after they have been irreversibly assigned to the participant.<br>This level of detail is rarely provided in reports, and a judgement may be required to justify an answer of 'Probably yes' or 'Probably no'.<br>'No' if there is reason to suspect that the enrolling investigator or the participant had knowledge of the forthcoming allocation.
**3) Did baseline differences between intervention groups suggest a problem with the randomization process?**	*Note that differences that are compatible with chance do not lead to a risk of bias. A small number of differences identified as 'statistically significant' at the conventional 0.05 threshold should usually be considered to be compatible with chance.*

	'No' if no imbalances are apparent or if any observed imbalances are compatible with chance.<br>'Yes' if there are imbalances that indicate problems with the randomization process, including: (1) substantial differences between intervention group sizes, compared with the intended allocation ratio; or (2) a substantial excess in statistically significant differences in baseline characteristics between intervention groups, beyond that expected by chance;<br>or (3) imbalance in one or more key prognostic factors, or baseline measures of outcome variables, that is very unlikely to be due to chance and for which the between-group difference is big enough to result in bias in the intervention effect estimate.<br>Also answer 'Yes' if there are other reasons to suspect that the randomization process was problematic: (4) excessive similarity in baseline characteristics that is not compatible with chance.<br><br>'No information' when there is no *useful* baseline information available (e.g. abstracts, or studies that reported only baseline characteristics of participants in the final analysis).<br>The answer to this question should not influence answers to questions 1.1 or 1.2. For example, if the trial has large baseline imbalances, but authors report adequate randomization methods, questions 1.1 and 1.2 should still be answered on the basis of the reported adequate methods, and any concerns about the imbalance should be raised in the answer to the question 1.3 and reflected in the domain-level risk-of-bias judgement.
**Risk-of-bias judgement**	Low / High / Some concerns
**Domain 2: Risk of bias due to deviations from the intended interventions (effect of assignment to intervention)**	
**1) Were participants aware of their assigned intervention during the trial?**	If participants are aware of their assigned intervention it is more likely that health-related behaviours will differ between the intervention groups. Blinding participants, most commonly through use of a placebo or sham intervention, may prevent such differences. If participants experienced side effects or toxicities that they knew to be specific to one of the interventions, answer this question 'Yes' or 'Probably yes'.
**2) Were carers and people delivering the interventions aware of participants' assigned intervention during the trial?**	If carers or people delivering the interventions are aware of the assigned intervention then its implementation, or administration of non-protocol interventions, may differ between the intervention groups. Blinding may prevent such differences. If participants experienced side effects or toxicities that carers or people delivering the interventions knew to be specific to one of the interventions, answer question 'Yes' or 'Probably yes'. If randomized allocation was not concealed, then it is likely that carers and people delivering the interventions were aware of participants' assigned intervention during the trial.
**3) If Y/PY/NI to 2.1 or 2.2: Were there deviations from the intended intervention that arose because of the trial context?**	For the effect of assignment to intervention, this domain assesses problems that arise when changes from assigned intervention that are inconsistent with the trial protocol arose because of the trial context. We use the term **trial context** to refer to effects of recruitment and engagement activities on trial participants and when trial personnel (carers or people delivering the interventions) undermine the implementation of the trial protocol in ways that would not happen outside the trial. For example, the process of securing informed consent may lead participants subsequently assigned to the comparator group to feel unlucky and therefore seek the experimental intervention, or other interventions that improve their prognosis.<br><br>Answer 'Yes' or 'Probably yes' **only** if there is evidence, or strong reason to believe, that the trial context led to failure to implement the protocol interventions or to implementation of interventions not allowed by the protocol.<br><br>Answer 'No' or 'Probably no' if there were changes from assigned

	intervention that are inconsistent with the trial protocol, such as non-adherence to intervention, but these are consistent with what could occur outside the trial context.<br><br>Answer 'No' or 'Probably no' for changes to intervention that are consistent with the trial protocol, for example cessation of a drug intervention because of acute toxicity or use of additional interventions whose aim is to treat consequences of one of the intended interventions.<br><br>If blinding is compromised because participants report side effects or toxicities that are specific to one of the interventions, answer 'Yes' or 'Probably yes' only if there were changes from assigned intervention that are inconsistent with the trial protocol and arose because of the trial context.<br>The answer 'No information' may be appropriate, because trialists do not always report whether deviations arose because of the trial context.
**4) If Y/PY to 2.3: Were these deviations likely to have affected the outcome?**	Changes from assigned intervention that are inconsistent with the trial protocol and arose because of the trial context will impact on the intervention effect estimate if they affect the outcome, but not otherwise.
**5) If Y/PY/NI to 2.4: Were these deviations from intended intervention balanced between groups?**	Changes from assigned intervention that are inconsistent with the trial protocol and arose because of the trial context are more likely to impact on the intervention effect estimate if they are not balanced between the intervention groups.
**6) Was an appropriate analysis used to estimate the effect of assignment to intervention?**	Both intention-to-treat (ITT) analyses and modified intention-to-treat (mITT) analyses excluding participants with missing outcome data should be considered appropriate. Both naïve 'per-protocol' analyses (excluding trial participants who did not receive their assigned intervention) and 'as treated' analyses (in which trial participants are grouped according to the intervention that they received, rather than according to their assigned intervention) should be considered inappropriate. Analyses excluding eligible trial participants post-randomization should also be considered inappropriate, but post- randomization exclusions of ineligible participants (when eligibility was not confirmed until after randomization, and could not have been influenced by intervention group assignment) can be considered appropriate.
**7) If N/PN/NI to 2.6: Was there potential for a substantial impact (on the result) of the failure to analyse participants in the group to which they were randomized?**	This question addresses whether the number of participants who were analysed in the wrong intervention group, or excluded from the analysis, was sufficient that there could have been a substantial impact on the result. It is not possible to specify a precise rule: there may be potential for substantial impact even if fewer than 5% of participants were analysed in the wrong group or excluded, if the outcome is rare or if exclusions are strongly related to prognostic factors.
**Risk-of-bias judgement**	Low / High / Some concerns
**Domain 2: Risk of bias due to deviations from the intended interventions (effect of adhering to intervention)**	
**1) Were participants aware of their assigned intervention during the trial?**	If participants are aware of their assigned intervention it is more likely that health-related behaviours will differ between the intervention groups. Blinding participants, most commonly through use of a placebo or sham intervention, may prevent such differences. If participants experienced side effects or toxicities that they knew to be specific to one of the interventions, answer this question 'Yes' or 'Probably yes'.

**2) Were carers and people delivering the interventions aware of participants' assigned intervention during the trial?**	If carers or people delivering the interventions are aware of the assigned intervention then its implementation, or administration of non-protocol interventions, may differ between the intervention groups. Blinding may prevent such differences. If participants experienced side effects or toxicities that carers or people delivering the interventions knew to be specific to one of the interventions, answer 'Yes' or 'Probably yes'. If randomized allocation was not concealed, then it is likely that carers and people delivering the interventions were aware of participants' assigned intervention during the trial.
**3) [If applicable:] If Y/PY/NI to 2.1 or 2.2: Were important non- protocol interventions balanced across intervention groups?**	This question is asked only if the preliminary considerations specify that the assessment will address imbalance of important non-protocol interventions between intervention groups. Important non-protocol interventions are the additional interventions or exposures that: (1) are inconsistent with the trial protocol; (2) trial participants might receive with or after starting their assigned intervention; and (3) are prognostic for the outcome. Risk of bias will be higher if there is imbalance in such interventions between the intervention groups.
**4) [If applicable:] Were there failures in implementing the intervention that could have affected the outcome?**	This question is asked only if the preliminary considerations specify that the assessment will address failures in implementing the intervention that could have affected the outcome. Risk of bias will be higher if the intervention was not implemented as intended by, for example, the health care professionals delivering care. Answer 'No' or 'Probably no' if implementation of the intervention was successful for most participants.
**5) [If applicable:] Was there non-adherence to the assigned intervention regimen that could have affected participants' outcomes?**	This question is asked only if the preliminary considerations specify that the assessment will address non- adherence that could have affected participants' outcomes. Non-adherence includes imperfect compliance with a sustained intervention, cessation of intervention, crossovers to the comparator intervention and switches to another active intervention. Consider available information on the proportion of study participants who continued with their assigned intervention throughout follow up, and answer 'Yes' or 'Probably yes' if the proportion who did not adhere is high enough to raise concerns. Answer 'No' for studies of interventions that are administered once, so that imperfect adherence is not possible, and all or most participants received the assigned intervention.
**6) If N/PN/NI to 2.3, or Y/PY/NI to 2.4 or 2.5: Was an appropriate analysis used to estimate the effect of adhering to the intervention?**	Both ' naïve 'per-protocol' analyses (excluding trial participants who did not receive their allocated intervention) and 'as treated' analyses (comparing trial participants according to the intervention they actually received) will usually be inappropriate for estimating the effect of adhering to intervention (the 'per-protocol' effect). However, it is possible to use data from a randomized trial to derive an unbiased estimate of the effect of adhering to intervention. Examples of appropriate methods include: (1) instrumental variable analyses to estimate the effect of receiving the assigned intervention in trials in which a single intervention, administered only at baseline and with all-or-nothing adherence, is compared with standard care; and (2) inverse probability weighting to adjust for censoring of participants who cease adherence to their assigned intervention, in trials of sustained treatment strategies. These methods depend on strong assumptions, which should be appropriate and justified if the answer to this question is 'Yes' or 'Probably yes'. It is possible that a paper reports an analysis based on such methods without reporting information on the deviations from intended intervention, but it would be hard to judge such an analysis to be appropriate in the absence of such information.<br><br>If an important non-protocol intervention was administered to all participants in one intervention group, adjustments cannot be made to overcome this.<br><br>Some examples of analysis strategies that would not be appropriate to estimate the effect of adhering to intervention are (i) 'Intention to treat (ITT) analysis', (ii) 'per protocol analysis', (iii) 'as-treated analysis',<br>(iv) 'analysis by treatment received'.

Risk-of-bias judgement	Low / High / Some concerns
**Domain 3: Risk of bias due to missing outcome data**	
**1) Were data for this outcome available for all, or nearly all, participants randomized?**	The appropriate study population for an analysis of the intention to treat effect is all randomized participants.<br>"Nearly all" should be interpreted as that the number of participants with missing outcome data is sufficiently small that their outcomes, whatever they were, could have made no important difference to the estimated effect of intervention.<br>For continuous outcomes, availability of data from 95% of the participants will often be sufficient. For dichotomous outcomes, the proportion required is directly linked to the risk of the event. If the observed number of events is much greater than the number of participants with missing outcome data, the bias would necessarily be small.<br>Only answer 'No information' if the trial report provides no information about the extent of missing outcome data. This situation will usually lead to a judgement that there is a high risk of bias due to missing outcome data.<br>Note that imputed data should be regarded as missing data, and not considered as 'outcome data' in the context of this question.
**2) If N/PN/NI to 3.1: Is there evidence that the result was not biased by missing outcome data?**	Evidence that the result was not biased by missing outcome data may come from: (1) analysis methods that correct for bias; or (2) sensitivity analyses showing that results are little changed under a range of plausible assumptions about the relationship between missingness in the outcome and its true value. However, imputing the outcome variable, either through methods such as 'last-observation-carried- forward' or via multiple imputation based only on intervention group, should not be assumed to correct for bias due to missing outcome data.
**3) If N/PN to 3.2: Could missingness in the outcome depend on its true value?**	If loss to follow up, or withdrawal from the study, could be related to participants' health status, then it is possible that missingness in the outcome was influenced by its true value. However, if all missing outcome data occurred for documented reasons that are unrelated to the outcome then the risk of bias due to missing outcome data will be low (for example, failure of a measuring device or interruptions to routine data collection).<br>In time-to-event analyses, participants censored during trial follow-up, for example because they withdrew from the study, should be regarded as having missing outcome data, even though some of their follow up is included in the analysis. Note that such participants may be shown as included in analyses in CONSORT flow diagrams.
**4) If Y/PY/NI to 3.3: Is it likely that missingness in the outcome depended on its true value?**	This question distinguishes between situations in which (i) missingness in the outcome could depend on its true value (assessed as 'Some concerns') from those in which (ii) it is likely that missingness in the outcome depended on its true value (assessed as 'High risk of bias'). Five reasons for answering 'Yes' are:<br>1. Differences between intervention groups in the proportions of missing outcome data. If there is a difference between the effects of the experimental and comparator interventions on the outcome, and the missingness in the outcome is influenced by its true value, then the proportions of missing outcome data are likely to differ between intervention groups. Such a difference suggests a risk of bias due to missing outcome data, because the trial result will be sensitive to missingness in the outcome being related to its true value. For time-to-event-data, the analogue is that rates of censoring (loss to follow-up) differ between the intervention groups.<br>2. Reported reasons for missing outcome data provide evidence that missingness in the outcome depends on

	its true value;<br>3. Reported reasons for missing outcome data differ between the intervention groups;<br>4. The circumstances of the trial make it likely that missingness in the outcome depends on its true value. For example, in trials of interventions to treat schizophrenia it is widely understood that continuing symptoms make drop out more likely.<br>5. In time-to-event analyses, participants' follow up is censored when they stop or change their assigned intervention, for example because of drug toxicity or, in cancer trials, when participants switch to second-line chemotherapy.<br>Answer 'No' if the analysis accounted for participant characteristics that are likely to explain the relationship between missingness in the outcome and its true value.
**Risk-of-bias judgement**	Low / High / Some concerns
**Domain 4: Risk of bias in measurement of the outcome**	
**1) Was the method of measuring the outcome inappropriate?**	This question aims to identify methods of outcome measurement (data collection) that are unsuitable for the outcome they are intended to evaluate. The question does not aim to assess whether the choice of outcome being evaluated was sensible (e.g. because it is a surrogate or proxy for the main outcome of interest). In most circumstances, for pre-specified outcomes, the answer to this question will be 'No' or 'Probably no'.<br>Answer 'Yes' or 'Probably yes' if the method of measuring the outcome is inappropriate, for example because:<br>(1) it is unlikely to be sensitive to plausible intervention effects (e.g. important ranges of outcome values fall outside levels that are detectable using the measurement method); or<br>(2) the measurement instrument has been demonstrated to have poor validity.
**2) Could measurement or ascertainment of the outcome have differed between intervention groups?**	Comparable methods of outcome measurement (data collection) involve the same measurement methods and thresholds, used at comparable time points. Differences between intervention groups may arise because of 'diagnostic detection bias' in the context of passive collection of outcome data, or if an intervention involves additional visits to a healthcare provider, leading to additional opportunities for outcome events to be identified.
**3) If N/PN/NI to 4.1 and 4.2: Were outcome assessors aware of the intervention received by study participants?**	Answer 'No' if outcome assessors were blinded to intervention status. For participant-reported outcomes, the outcome assessor is the study participant.
**4) If Y/PY/NI to 4.3: Could assessment of the outcome have been influenced by knowledge of intervention received?**	Knowledge of the assigned intervention could influence participant-reported outcomes (such as level of pain), observer-reported outcomes involving some judgement, and intervention provider decision outcomes. They are unlikely to influence observer-reported outcomes that do not involve judgement, for example all-cause mortality.
**5) If Y/PY/NI to 4.4: Is it likely that assessment of the outcome was influenced by knowledge of intervention received?**	This question distinguishes between situations in which (i) knowledge of intervention status could have influenced outcome assessment but there is no reason to believe that it did (assessed as 'Some concerns') from those in which (ii) knowledge of intervention status was likely to influence outcome assessment (assessed as 'High'). When there are strong levels of belief in either beneficial or harmful effects of the intervention, it is more likely that the outcome was influenced by knowledge of the intervention received. Examples may include patient-reported

	symptoms in trials of homeopathy, or assessments of recovery of function by a physiotherapist who delivered the intervention.
**Risk-of-bias judgement**	Low / High / Some concerns
**Domain 5: Risk of bias in selection of the reported result**	
**1) Were the data that produced this result analysed in accordance with a pre-specified analysis plan that was finalized before unblinded outcome data were available for analysis?**	If the researchers' pre-specified intentions are available in sufficient detail, then planned outcome measurements and analyses can be compared with those presented in the published report(s). To avoid the possibility of selection of the reported result, finalization of the analysis intentions must precede availability of unblinded outcome data to the trial investigators.<br>Changes to analysis plans that were made before unblinded outcome data were available, or that were clearly unrelated to the results (e.g. due to a broken machine making data collection impossible) do not raise concerns about bias in selection of the reported result.
**2) Is the numerical result being assessed likely to have been selected, on the basis of the results, from multiple eligible outcome measurements (e.g. scales, definitions, time points) within the outcome domain?**	Answer 'Yes' or 'Probably yes' if: There is clear evidence (usually through examination of a trial protocol or statistical analysis plan) that a domain was measured in multiple eligible ways, but data for only one or a subset of measures is fully reported (without justification), and the fully reported result is likely to have been selected on the basis of the results. Selection on the basis of the results can arise from a desire for findings to be newsworthy, sufficiently noteworthy to merit publication, or to confirm a prior hypothesis. For example, trialists who have a preconception, or vested interest in showing, that an experimental intervention is beneficial may be inclined to report outcome measurements selectively that are favourable to the experimental intervention.<br><br>Answer 'No' or 'Probably no' if: There is clear evidence (usually through examination of a trial protocol or statistical analysis plan) that all eligible reported results for the outcome domain correspond to all intended outcome measurements.<br>Or There is only one possible way in which the outcome domain can be measured (hence there is no opportunity to select from multiple measures).<br>Or Outcome measurements are inconsistent across different reports on the same trial, but the trialists have provided the reason for the inconsistency and it is not related to the nature of the results.<br><br>Answer 'No information' if: Analysis intentions are not available, or the analysis intentions are not reported in sufficient detail to enable an assessment, and there is more than one way in which the outcome domain could have been measured.
**3) Is the numerical result being assessed likely to have been selected, on the basis of the results, from multiple eligible analyses of the data?**	Answer 'Yes' or 'Probably yes' if: There is clear evidence (usually through examination of a trial protocol or statistical analysis plan) that a measurement was analysed in multiple eligible ways, but data for only one or a subset of analyses is fully reported (without justification), and the fully reported result is likely to have been selected on the basis of the results. Selection on the basis of the results arises from a desire for findings to be newsworthy, sufficiently noteworthy to merit publication, or to confirm a prior

	hypothesis. For example, trialists who have a preconception or vested interest in showing that an experimental intervention is beneficial may be inclined to selectively report analyses that are favourable to the experimental intervention.<br><br>Answer 'No' or 'Probably no' if: There is clear evidence (usually through examination of a trial protocol or statistical analysis plan) that all eligible reported results for the outcome measurement correspond to all intended analyses.<br>Or There is only one possible way in which the outcome measurement can be analysed (hence there is no opportunity to select from multiple analyses).<br>Or Analyses are inconsistent across different reports on the same trial, but the trialists have provided the reason for the inconsistency and it is not related to the nature of the results.<br><br>Answer 'No information' if: Analysis intentions are not available, or the analysis intentions are not reported in sufficient detail to enable an assessment, and there is more than one way in which the outcome measurement could have been analysed.
**Risk-of-bias judgement**	Low / High / Some concerns
**Overall risk-of-bias judgement**	
**Risk-of-bias judgement**	Low risk of bias: the study is judged to be at **low risk of bias for all domains** for this result.<br><br>Some concerns: the study is judged to raise **some concerns** in at least one domain for this result, but not to be at high risk of bias for any domain.<br><br>High risk of bias: The study is judged to be at **high risk of bias** in at least one domain for this result. Or the study is judged to have **some concerns** for **multiple domains** in a way that substantially lowers confidence in the result.

FIGURES

Figure 1. Number of Covid-19-related medical articles of different types of publication. (A) All; (B) Peer-reviewed articles; (C) Preprints.

A

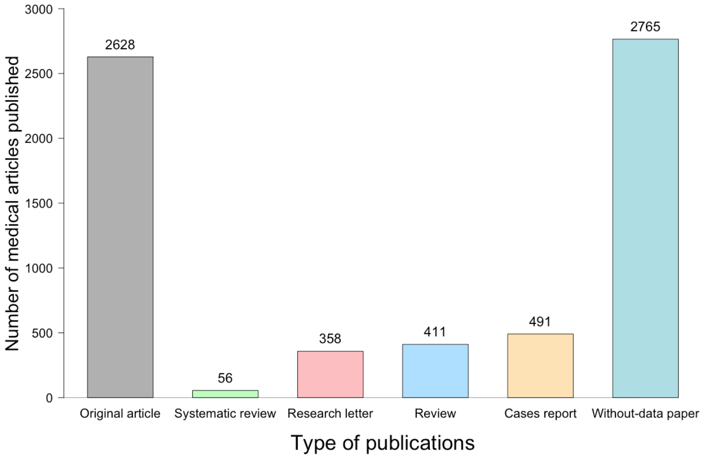

B

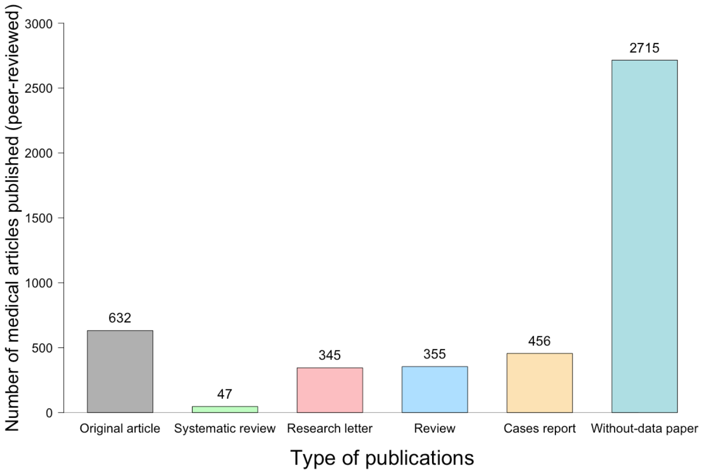

C

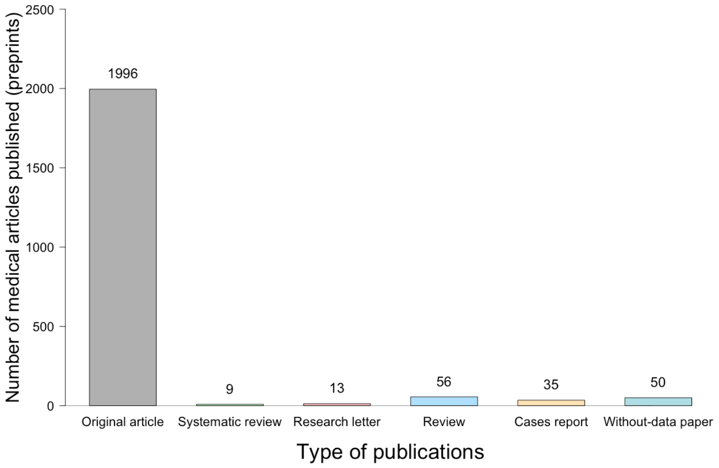

Figure 2. Dynamics of the accumulated number of Covid-19-related medical article since 2019-11-01: preprints vs peer-reviewed articles.

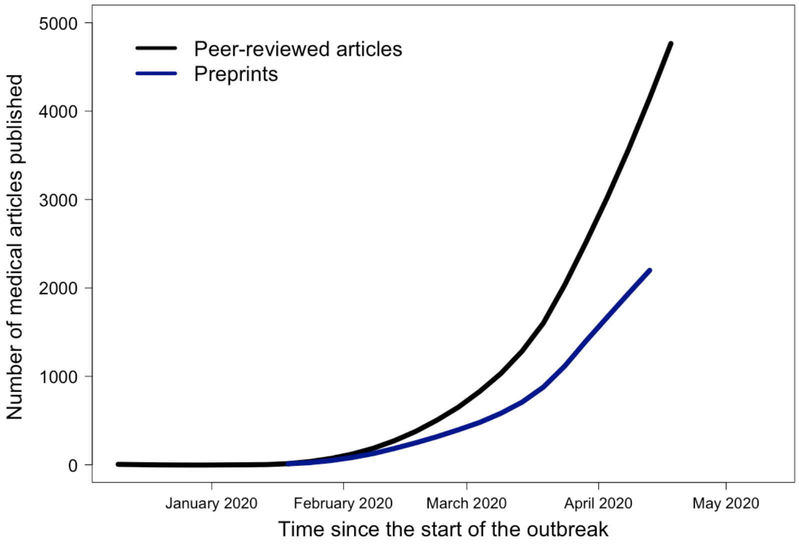

Figure 3. Dynamics of the accumulated number of Covid-19-related medical article stratified by top 10 productive countries. (A) China; (B) United States; (C) United Kingdom; (D) Italy; (E) India; (F) France; (G) Canada; (H) Germany; (I) Australia; (J) Iran.

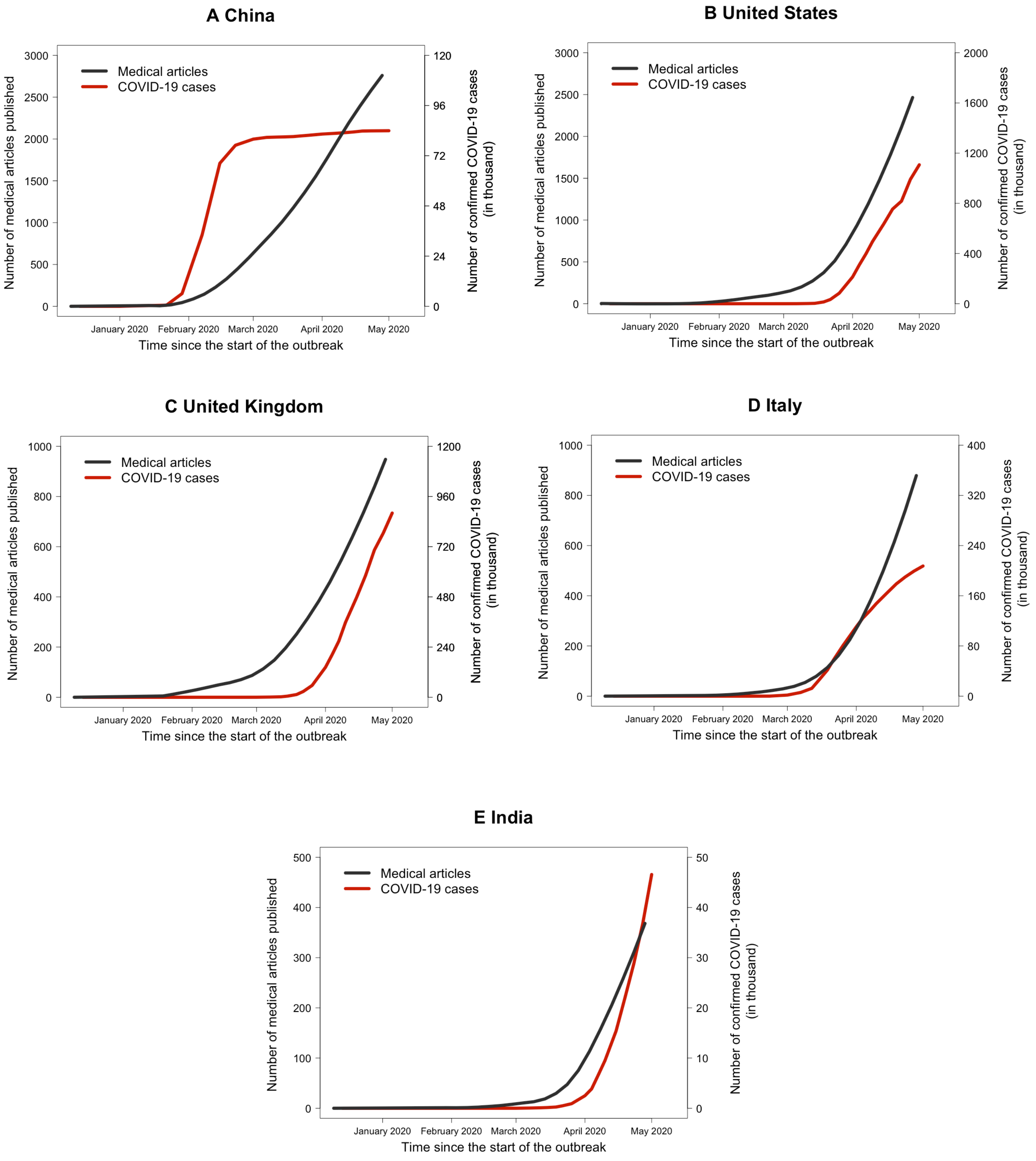

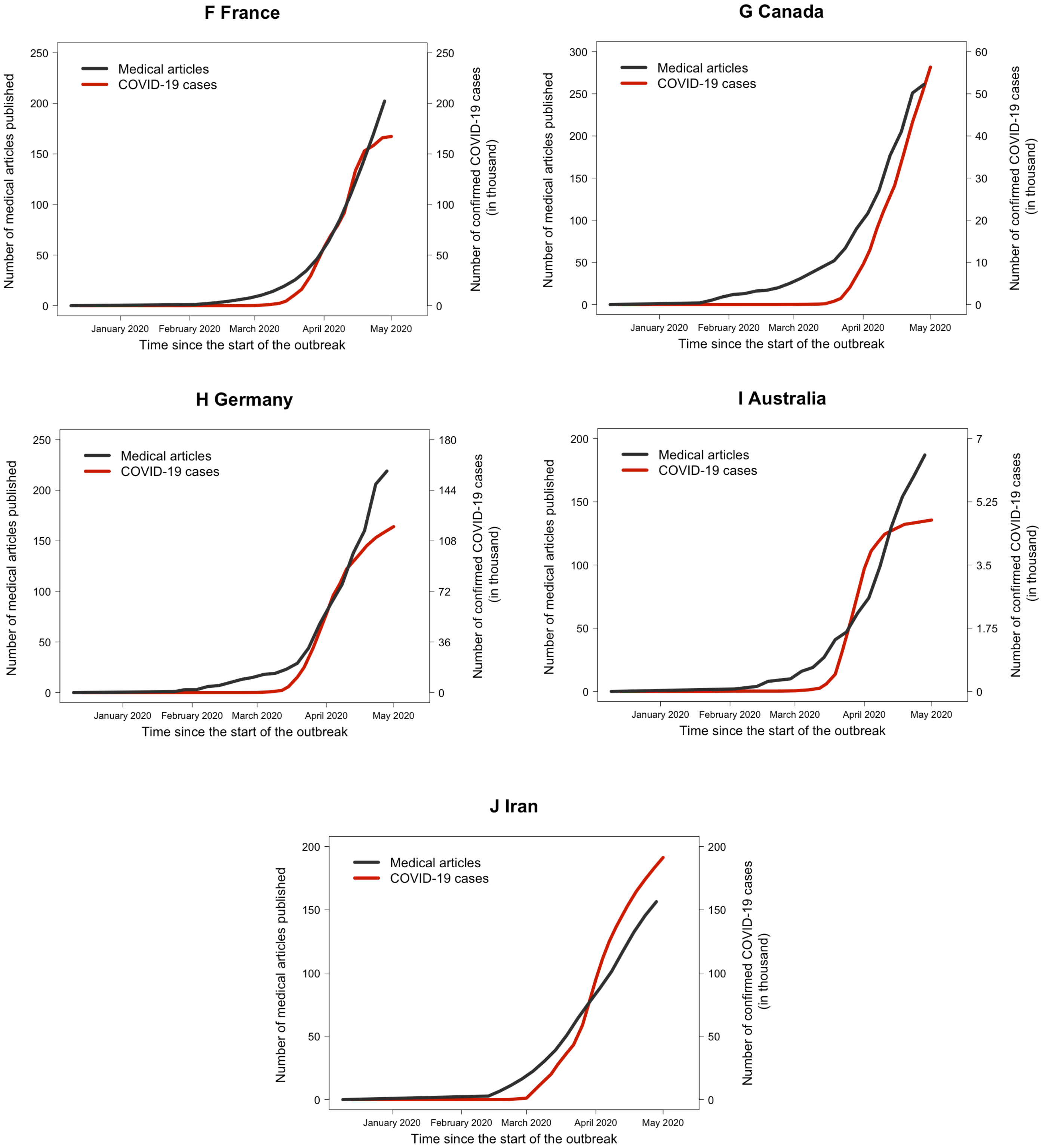
F France
Medical articles
COVID-19 cases
Number of medical articles published
Number of confirmed COVID-19 cases (in thousand)
0 50 100 150 200 250
January 2020 February 2020 March 2020 April 2020 May 2020
Time since the start of the outbreak
G Canada
Medical articles
COVID-19 cases
Number of medical articles published
0 50 100 150 200 250 300
Number of confirmed COVID-19 cases (in thousand)
0 10 20 30 40 50 60
January 2020 February 2020 March 2020 April 2020 May 2020
Time since the start of the outbreak
H Germany
Medical articles
COVID-19 cases
Number of medical articles published
0 50 100 150 200 250
Number of confirmed COVID-19 cases (in thousand)
0 36 72 108 144 180
January 2020 February 2020 March 2020 April 2020 May 2020
Time since the start of the outbreak
I Australia
Medical articles
COVID-19 cases
Number of medical articles published
0 50 100 150 200
Number of confirmed COVID-19 cases (in thousand)
0 1.75 3.5 5.25 7
January 2020 February 2020 March 2020 April 2020 May 2020
Time since the start of the outbreak
J Iran
Medical articles
COVID-19 cases
Number of medical articles published
0 50 100 150 200
Number of confirmed COVID-19 cases (in thousand)
0 50 100 150 200
January 2020 February 2020 March 2020 April 2020 May 2020
Time since the start of the outbreak

Figure 4. Topic distribution of COVID-19-related, peer-reviewed medical articles

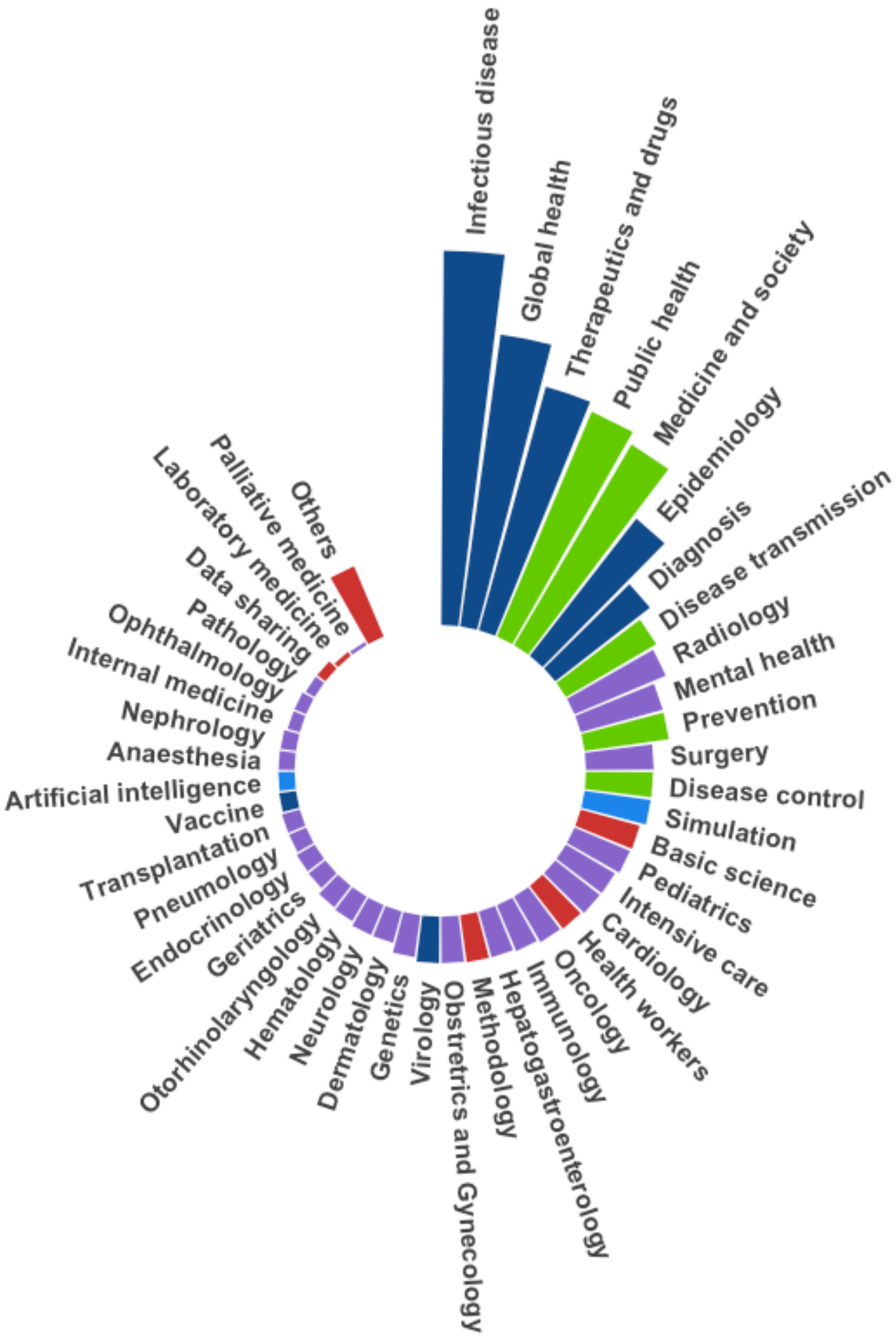

Figure 5. Topic distribution of COVID-19-related, preprint medical articles

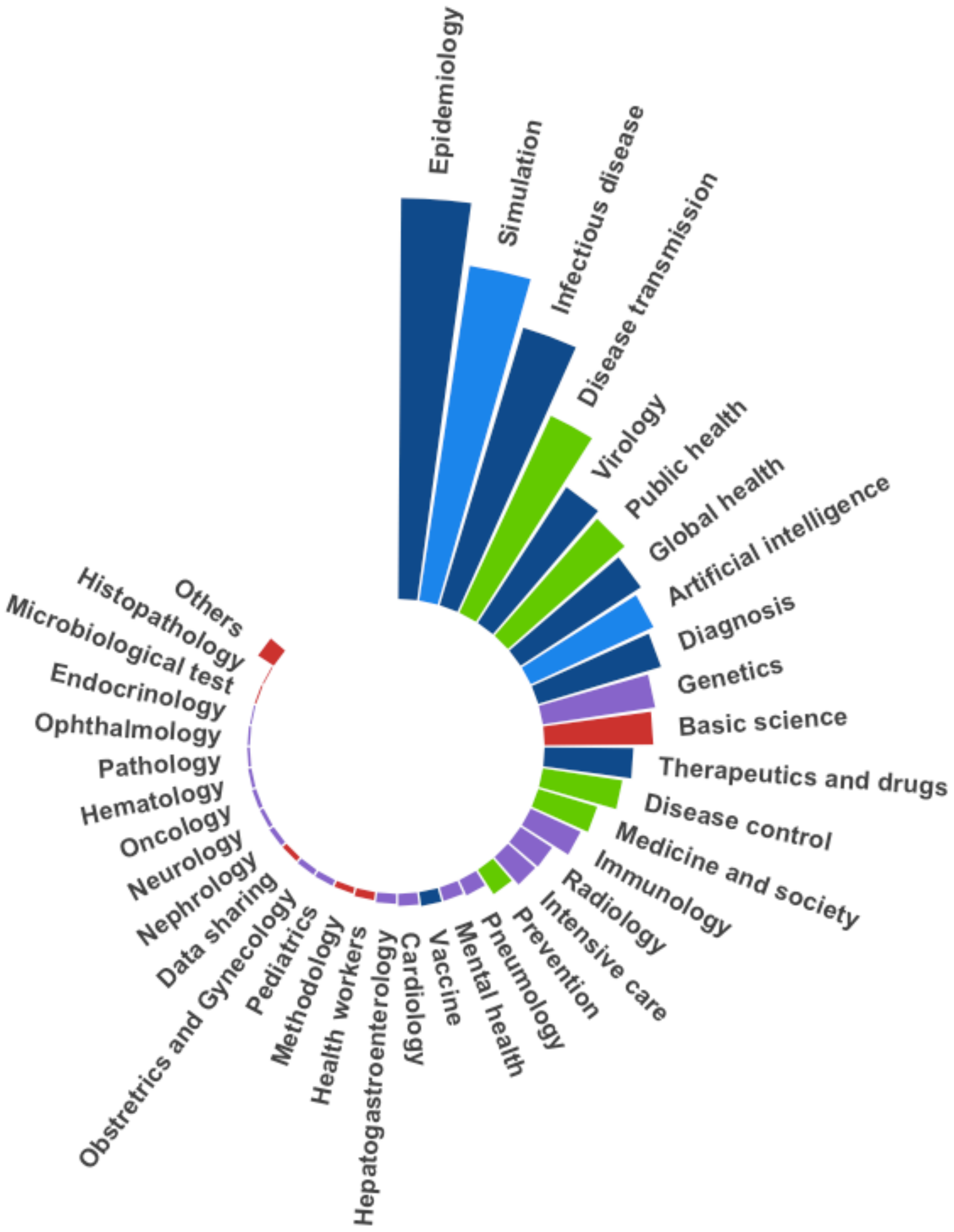

Figure 6- Assessment of the quality of research in peer-reviewed original articles

713 clinical, peer-reviewed, COVID-19-related, original articles were critically appraised based on several risk of bias tools (supplementary methods). Original articles were categorized into several study design: case-control studies, cohort studies, cross-sectional studies, original articles with case series data, diagnostic studies, prognostic studies, simulation-based studies, non-randomized interventional studies, and randomized controlled trials (table 1 for more details). The three latter, least represented, are detailed in the supplementary figures 7, 8 and 9. Each line represents one study. **Green** cases indicate that authors adequately addressed the corresponding items. **Red** cases indicate that authors did not adequately address the corresponding items. **Black** cases indicate that the item was not applicable to the study design.

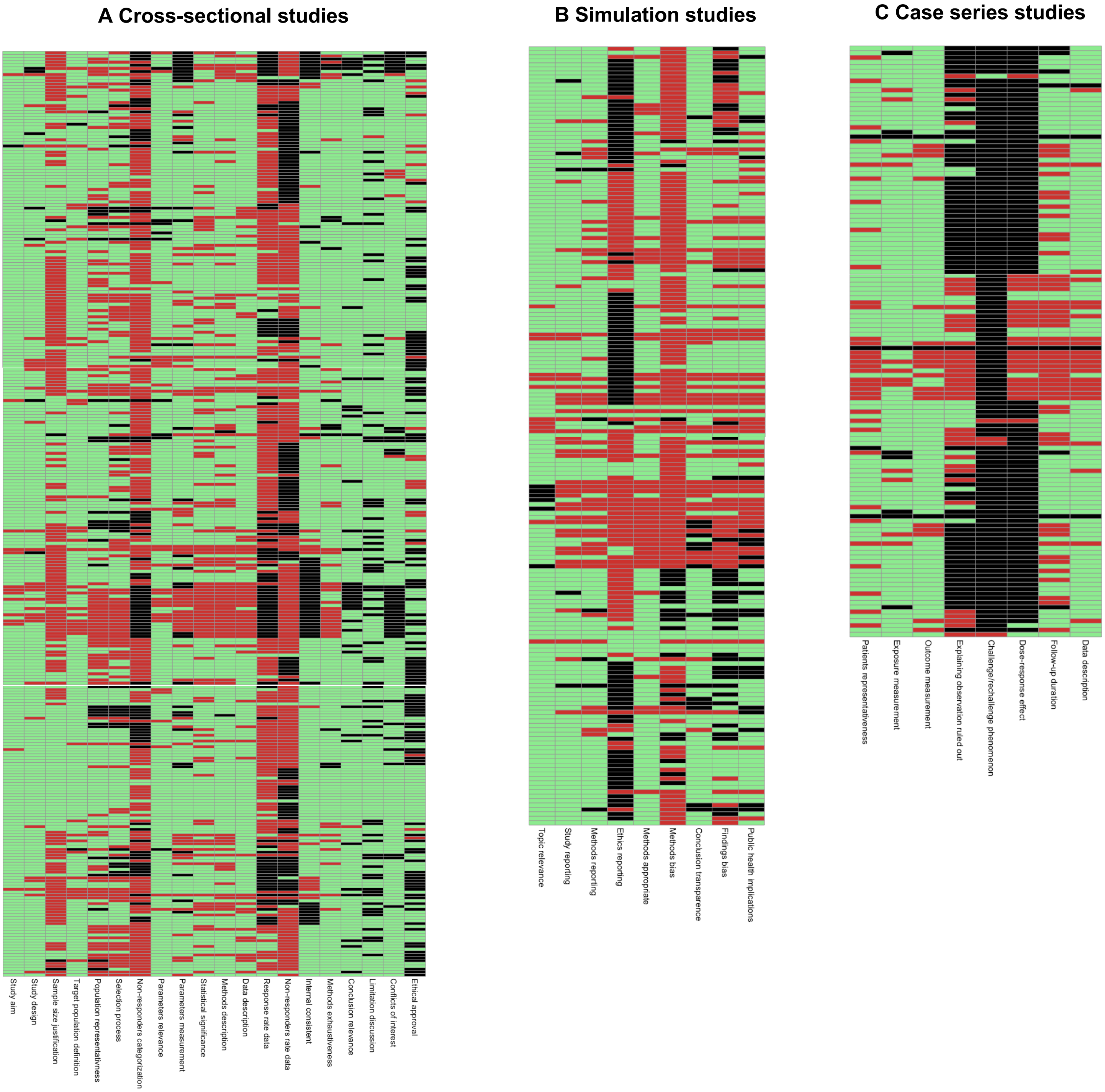

E Case-control studies

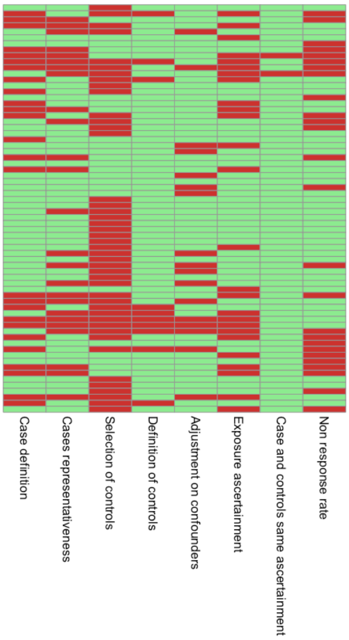

F Cohort studies

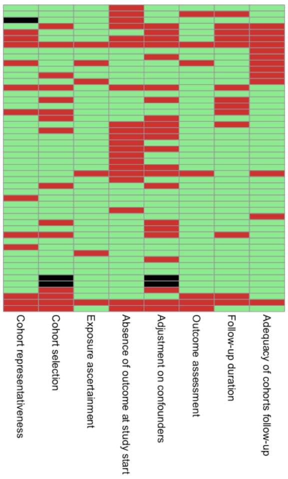

G Diagnostic studies

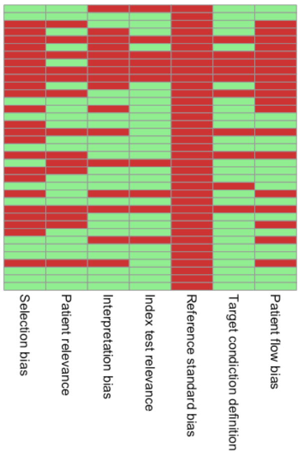

Figure 7. Heatmap showing appraisal results of prognostic studies. Green represents the items adequately addressed; **Red** represents the items not adequately addressed; **Black** represents the items not applicable.

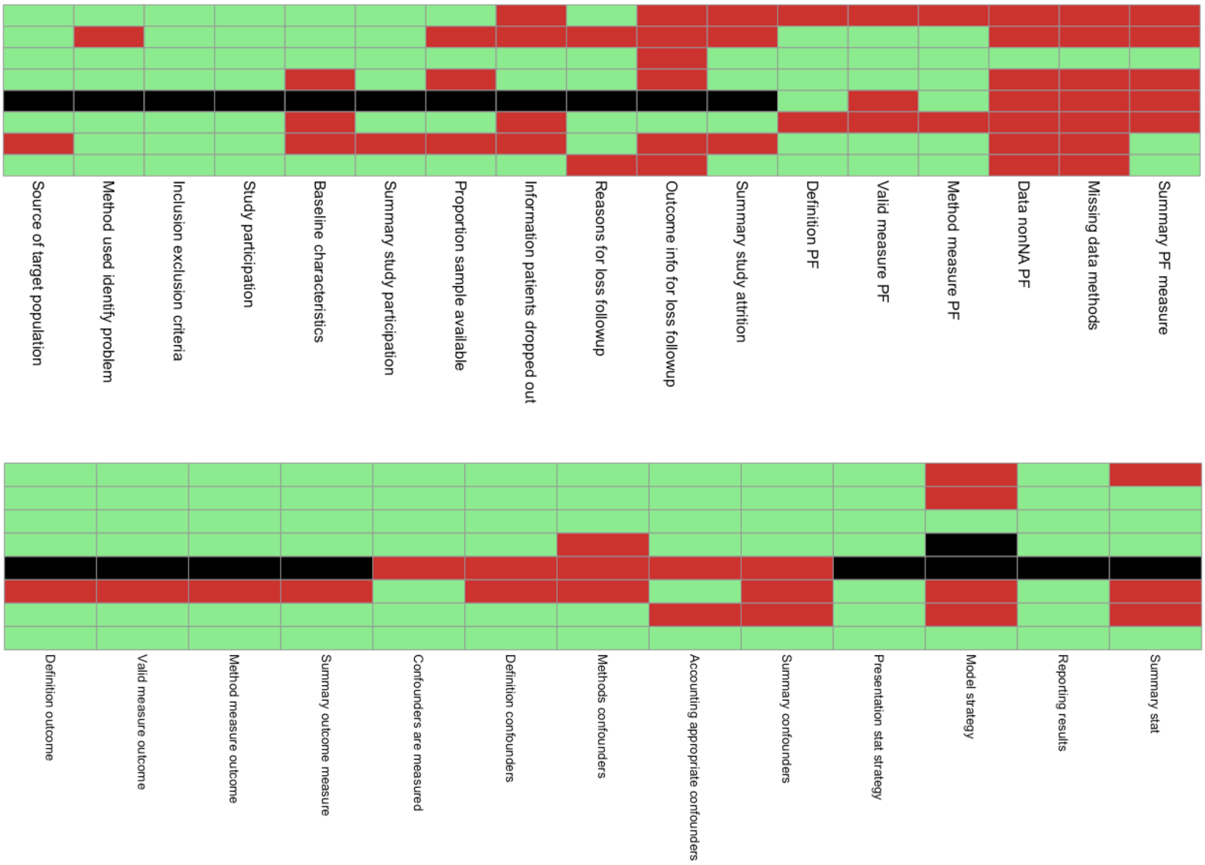

Figure 8. Heatmap showing appraisal results of non-randomised interventional studies. Green represents the items adequately addressed; **Red** represents the items not adequately addressed; **Black** represents the items not applicable.

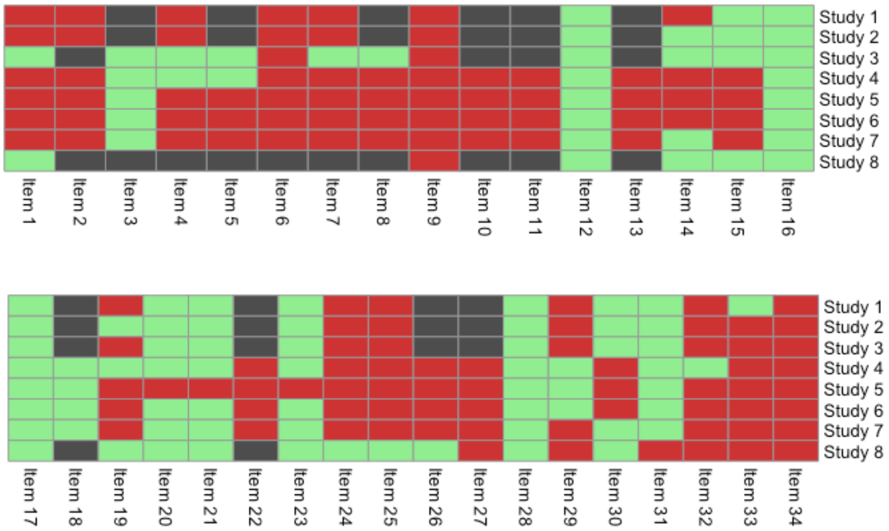

Figure 9. Heatmap showing appraisal results of randomised controlled studies. Green represents the items adequately addressed; **Red** represents the items not adequately addressed; **Black** represents the items not applicable.

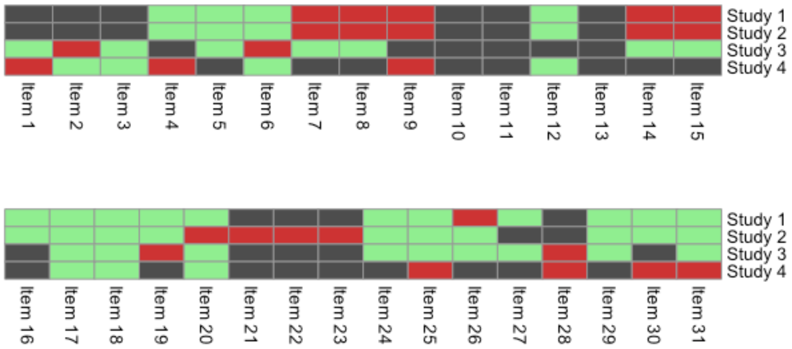

Figure 10. Adequacy in the risk of bias for the 82 studies evaluated with two assessment tools.

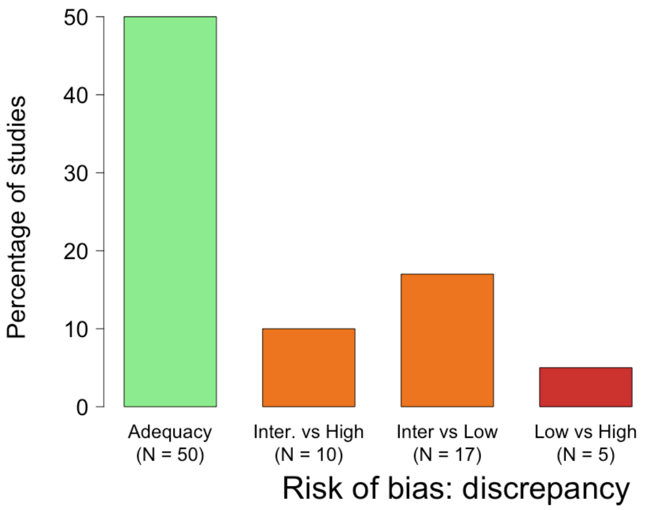

References

1. Rosella L, Bowman C, Pach B, Morgan S, Fitzpatrick T, Goel V. The development and validation of a meta-tool for quality appraisal of public health evidence: Meta Quality Appraisal Tool (MetaQAT). Public health 2016;136:57-65.
2. Downes MJ, Brennan ML, Williams HC, Dean RS. Development of a critical appraisal tool to assess the quality of cross-sectional studies (AXIS). BMJ open 2016;6:e011458.
3. Murad MH, Sultan S, Haffar S, Bazerbachi F. Methodological quality and synthesis of case series and case reports. BMJ evidence-based medicine 2018;23:60-3.
4. The Newcastle-Ottawa Scale (NOS) for assessing the quality of nonrandomised studies in meta-analyses. at http://www.ohri.ca/programs/clinical_epidemiology/oxford.asp.)
5. Whiting PF, Rutjes AW, Westwood ME, et al. QUADAS-2: a revised tool for the quality assessment of diagnostic accuracy studies. Annals of internal medicine 2011;155:529-36.
6. Hayden JA, van der Windt DA, Cartwright JL, Côté P, Bombardier C. Assessing bias in studies of prognostic factors. Annals of internal medicine 2013;158:280-6.
7. Sterne JA, Hernan MA, Reeves BC, et al. ROBINS-I: a tool for assessing risk of bias in non-randomised studies of interventions. BMJ (Clinical research ed) 2016;355:i4919.
8. Sterne JAC, Savovic J, Page MJ, et al. RoB 2: a revised tool for assessing risk of bias in randomised trials. BMJ (Clinical research ed) 2019;366:l4898.
9. Kempf E, de Beyer JA, Cook J, et al. Overinterpretation and misreporting of prognostic factor studies in oncology: a systematic review. British journal of cancer 2018;119:1288-96.
10. Ji W, Wang W, Zhao X, Zai J, Li X. Cross-species transmission of the newly identified coronavirus 2019-nCoV. Journal of medical virology 2020;92:433-40.
